# Mentoring und arbeitsplatzbasierte Prüfungen im Praktischen Jahr: Ein effektiver Weg zur Steigerung von Zufriedenheit und Kompetenz?

**DOI:** 10.1007/s00101-020-00902-7

**Published:** 2020-12-14

**Authors:** A. Weissenbacher, R. Bolz, A. Zimmermann, B. Donaubauer, S. N. Stehr, G. Hempel

**Affiliations:** 1 Klinik und Poliklinik für Anästhesiologie und Intensivtherapie, Universitätsklinikum Leipzig AöR, Leipzig, Deutschland; 2 Lernklinik Leipzig – Skills- und Simulationszentrum, Universität Leipzig, Leipzig, Deutschland

**Keywords:** Medizindidaktik, Curriculumsentwicklung, Anästhesiologie, Medizinstudium, Kompetenzerwerb, Medical education, Curriculum development, Anesthesiology, Medical studies, Competence

## Abstract

**Hintergrund:**

Das praktische Jahr (PJ) ist an vielen Universitäten der am wenigsten strukturierte und standardisierte Studienabschnitt. Studierende beklagen mangelnde Anleitung, Supervision und Feedback. Häufig übernehmen sie delegationsfähige, nichtmedizinische Aufgaben, obwohl das PJ ein Hauptentscheidungsfaktor für die spätere Facharztwahl ist.

**Methoden:**

Nach einer Bedarfsanalyse erfolgte die Entwicklung eines Mentoring-basierten Curriculums für Studierende im PJ mithilfe des Kern-Zyklus. Hierzu wurden 10 fachspezifische klinisch-praktische Basiskompetenzen etabliert, die jeder Studierende bis zum Tertialende beherrschen sollte. Eine Überprüfung erfolgte formativ anhand von arbeitsplatzbasierten Prüfungen. Das Tertial wurde durch alle Studierenden abschließend online evaluiert.

**Ergebnisse:**

Die Priorisierung und Prüfung von klinisch-praktischen Kompetenzen durch Mentoren/Mentorinnen ermöglichten eine bedarfsorientierte und qualitative hochwertige Ausbildung. Das Mentoring und Feedback wurden durchweg positiv beurteilt und unterstützten den Lernerfolg (Note 1,5). Das Prüfungsformat wurde mehrheitlich als unbekannt (64,6 %), aber hilfreich und sinnvoll erlebt (76,7 %). Studierende fühlten sich durch das Curriculum gut auf die Staatsexamensprüfung (81,3 %) und den Berufsbeginn vorbereitet (71,0 %). Dies ging mit einer hohen Zufriedenheit (Note 1,7) einher.

**Schlussfolgerungen:**

Ein bedarfsgerechtes, Mentoring-basiertes Curriculum mit integrierten arbeitsplatzbasierten Prüfungen geht nicht nur mit einer hohen Ausbildungszufriedenheit einher, sondern fördert effektiv und ressourcenschonend die Ausbildungsqualität.

**Zusatzmaterial online:**

Die Online-Version dieses Beitrags (10.1007/s00101-020-00902-7) enthält das Prüfungsformat, den Fragebogen und die Evaluationsdaten. Beitrag und Zusatzmaterial stehen Ihnen auf www.springermedizin.de zur Verfügung. Bitte geben Sie dort den Beitragstitel in die Suche ein, das Zusatzmaterial finden Sie beim Beitrag unter „Ergänzende Inhalte“.
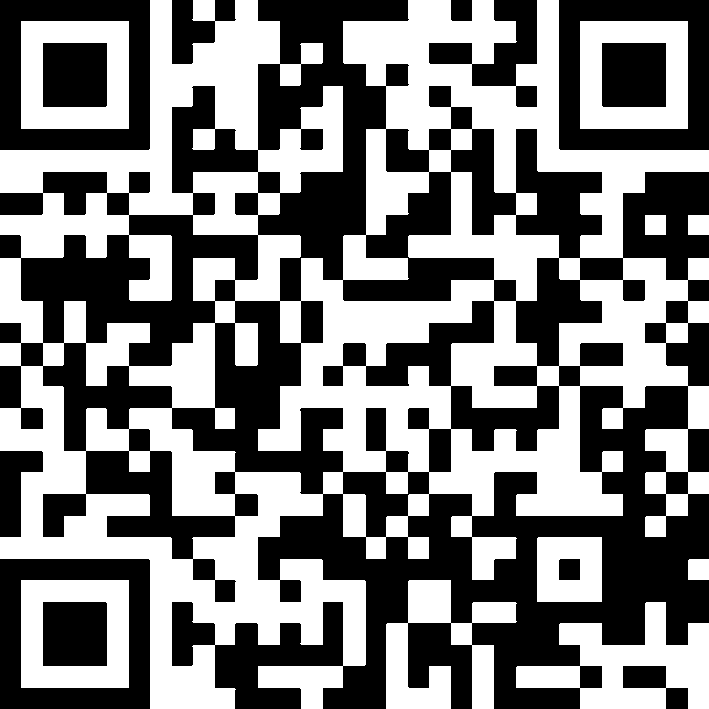

## Abstract (en)

**Background:**

The final year of medical training in Germany is one of the least structured and standardized years of medical school. Medical students often complain about a lack of guidance, supervision and feedback. They are mostly asked to perform delegable nonmedical tasks even though student experiences in this period critically determine future decisions for certain medical specialties. Consequently, right from the beginning many young professionals feel overburdened especially by the time pressure of everyday clinical practice. The planned amendment of the medical licensing regulations will make competence-based training even more important. This article therefore aims to examine the extent to which a mentoring-based curriculum with workplace-based examinations during the final year of medical studies can make a valuable contribution to this.

**Methods:**

After a needs assessment (structured literature search, results evaluation and focus groups with both students and medical specialists), a mentoring-based curriculum for final year medical students was developed following the Kern cycle. In 2 work sessions 10 discipline-specific competencies for the fields of anesthesiology, critical care, emergency and pain medicine were established and prioritized, which had to be mastered by every student independently at the end of the training period. Assessment of these competencies was performed on a regular basis by trained mentors in the form of workplace-based assessments (mini-clinical evaluation exercise, mini-CEX, direct observation of procedural skills, DOPS). Multiperspective evaluation was and is the foundation of continuous program development. By September 2019 a total of 40 students had completed the modified curriculum and were subsequently interviewed online about various aspects of the tertial.

**Results:**

The response rate to the survey was 80% (*n* = 32). The gender ratio was balanced (male = 50%, female = 50%). Prioritization and assessment of 10 competencies by trained mentors enabled a focused, demand-driven and high-quality training of final year medical students. Surveyed students found the section mentoring and feedback to be very positive and it supported their learning success (grade 1.5). Despite firmly established feedback structures, in retrospect almost half (51.6%) wanted more structured feedback. Workplace-based assessments were mostly previously unknown (64.6%) but were experienced as helpful and meaningful (76.7%). Students felt confident and prepared for the final state examination (81.3%) and their career start (71.0%) after being part of the program. These findings were accompanied by a high level of satisfaction (grade 1.7) as well as a high recommendation rate for this institution (as a training program for final year medical students and as a career start for residents, both with 93.7%). Thus, the good evaluation results of the department before the start of the project could again be slightly improved.

**Conclusion:**

A demand-driven, mentoring-based curriculum with integrated workplace-based assessments not only led to high overall student satisfaction but also promoted the quality of teaching in an effective and resource-saving way. Mentoring promotes learning success mainly through feedback and individual learning support and also supports the communicative and social skills of students and mentors alike.

## Hinführung zum Thema

Mit der Veröffentlichung des Masterplans 2020 durch das Bundesministerium für Bildung und Forschung (BMBF) und des neuen Gegenstandskatalogs durch das Institut für medizinische und pharmazeutische Prüfungsfragen (IMPP) rückt die kompetenzbasierte medizinische Ausbildung endgültig in den Mittelpunkt des Humanmedizinstudiums.

Mit der folgenden Arbeit sollte daher untersucht werden, inwieweit ein Mentoring-basiertes Curriculum mit arbeitsplatzbasierten Prüfungen im praktischen Jahr hierzu einen wertvollen Beitrag leisten kann.

## Hintergrund

Jährlich werden im Rahmen des Humanmedizinstudiums in Deutschland ca. 12.000 Studierende im sog. praktischen Jahr (PJ) – dem letzten Studienabschnitt – ausgebildet. Ziele des PJ sind die Anwendung des zuvor im Studium erlangten theoretischen Wissens im klinischen Alltag sowie das Trainieren von praktischen ärztlichen Tätigkeiten im realen Arbeitsumfeld. Hier sollen professionelle Haltungen, Fähigkeiten und Fertigkeiten, die essenziell für den Berufsstart als approbierte Ärztinnen und Ärzte sind, in einer möglichst hohen Kompetenzstufe erworben werden.

Die Fokussierung auf eine kompetenzbasierte Ausbildung im Medizinstudium wurde bereits 2014 vom Wissenschaftsrat empfohlen und die Umsetzung an den Fakultäten durch das Bundesministerium für Bildung und Forschung (BMBF) mit dem Masterplan Medizinstudium 2020 zur Verbesserung der universitären Lehre gefordert [27, 28]. Sowohl der Nationale Kompetenzbasierte Lernzielkatalog Medizin (NKLM) als auch der Lernzielkatalog der Deutschen Gesellschaft für Anästhesiologie und Intensivmedizin (DGAI) sind umfangreiche Verzeichnisse, die für eine Lernzieldefinition herangezogen werden können. Die erarbeiteten Lernziele sind entsprechend § 3 der derzeitigen Ärztlichen Approbationsordnung (ÄApprO) in einem Ausbildungsplan für Studierende im PJ unter Verwendung von Logbüchern zu hinterlegen [3, 11]. Weiter ist der NKLM Grundlage für die aktuelle Ausarbeitung des neuen Gegenstandskatalogs, auf dessen Basis zukünftig auch klinisch-praktische Kompetenzen standardisiert im abschließenden Staatsexamen geprüft werden sollen [15].

Das PJ ist bis dato an vielen medizinischen Fakultäten der am wenigsten strukturierte und standardisierte Studienabschnitt. Studierende beklagen nicht nur fehlende oder mangelnde Anleitung und Supervision, sondern ebenso nichtausreichendes Feedback im Rahmen ihrer klinischen Tätigkeit [22]. Vielmehr ist es nicht unüblich, dass Studierende nur eine Beobachterrolle einnehmen und für unliebsame, delegationsfähige und nichtmedizinische Tätigkeiten herangezogen werden, die nicht oder nur in sehr geringem Maße die Ausbildung fördern [29]. Somit wird eine vollständige Teamintegration verpasst. Diese Situation ist für die Studierenden und die betreuenden ärztlichen Kolleginnen und Kollegen oft gleichermaßen frustrierend. Repräsentative Erhebungen bestätigten, dass sich zum Weiterbildungsbeginn Ärztinnen und Ärzte nicht nur fachlich überfordert, sondern ebenfalls durch ihre Tätigkeit überlastet und einem hohem Zeitdruck ausgesetzt fühlen [12]. Zudem ist evident, dass bereits im Medizinstudium und auch im Rahmen des Berufsstarts, aufgrund von Stress und Überforderung, das Risiko für das Auftreten von Depression, Burn-out, Angststörungen und Substanzabhängigkeit überdurchschnittlich erhöht ist [4, 9, 14].

Die klinischen Erfahrungen im PJ sind der wichtigste Entscheidungsfaktor für die Facharztwahl [12]. Ein Mentoring-basiertes Ausbildungskonzept kann nicht nur die Facharztwahl beeinflussen [8], sondern unterstützt ebenso positiv akademische Leistung, Sozialkompetenz und Stressverarbeitung [7, 17]. Darüber hinaus sind Kompetenzerleben, Autonomie und soziale Eingebundenheit Faktoren, die die intrinsische Lernmotivation von Studierenden fördern können [6]. Alle drei Faktoren sollten durch ein Mentoring-basiertes Ausbildungsprogramm im Rahmen des PJ positiv unterstützt werden, um so den Lernerfolg zu maximieren [5]. Mentoring ermöglicht es, höhere Kompetenzebenen, Selbstständigkeit und Exzellenz durch „deliberate practice“ – ein systematisches und zielgerichtetes Ausbildungskonzept – zu erreichen [10]. Es schafft im Rahmen einer 1:1-Betreuung nicht nur exzellente Ausbildungsverhältnisse für Studierende, sondern das Lehren und die Betreuung selbst erhöhen bei den ärztlichen Mentorinnen und Mentoren Selbstsicherheit, Reflexionsfähigkeit, klinische, kommunikative sowie lehrbezogene Fähig- und Fertigkeiten [21, 23, 26].

Strukturiertes Feedback – als essenzieller Bestandteil eines guten Mentorings – ist nachweislich eine der effektivsten Methoden in der medizinischen Ausbildung, um eine Verhaltensänderung der Lernenden herbeizuführen [13, 24]. Arbeitsplatzbasierte, formative Prüfungsformate wie das *Mini Clinical Evaluation Exercise* (Mini-CEX) oder *Direct Observation of Procedural Skills* (DOPS) sind bei wiederholter Anwendung validierte und standardisierte Formate, die dies Outcome-orientiert zur Anwendung bringen [19]. Bei beiden Prüfungsformaten handelt es sich um checklistenbasierte Werkzeuge, die es Feedbackgebenden erlauben, die Durchführung der jeweiligen Lernziele standardisiert zu beurteilen und, darauf stützend, das sich obligat anschließende Feedback zu strukturieren.

An der Medizinischen Fakultät der Universität Leipzig wurde daher im Rahmen des PJ-Tertials „Anästhesiologie“ am Universitätsklinikum Leipzig (UKL) ein neues Mentoring-basiertes Curriculum mit regelmäßigen arbeitsplatzbasierten Prüfungen zur Verbesserung der Lehre und insbesondere des Kompetenzerwerbs etabliert und kontinuierlich evaluiert.

## Methoden

Es wurde ein neues Mentoring-basiertes Ausbildungscurriculum für das PJ-Tertial „Anästhesiologie“ nach dem Kernmodell entwickelt [16]. Der Kern-Zyklus beschreibt ein 6‑stufiges, dynamisches Modell zur Entwicklung medizinischer Curricula, das Grundprinzipien eines Qualitätsmanagements mit dem Ziel der kontinuierlichen Verbesserung entspricht [25]. Einer allgemeinen Bedarfsanalyse folgt dabei immer eine spezifische Analyse der Zielgruppe als Grundlage für die Definition von Lernzielen. Nachdem für diese Lernziele passende Lehrmethoden festgelegt wurden, erfolgen im nächsten Schritt die Implementierung und letztlich die regelmäßige Evaluation und kontinuierliche Weiterentwicklung des Curriculums. Im Zuge der Entwicklung der Lernziele erfolgte in zwei klinikinternen Lehrkonferenzen mit einer Gruppe von ärztlichen Kollegen/Kolleginnen, die an der PJ-Ausbildung beteiligt sind, eine Definition und Priorisierung von insgesamt 10 klinisch-praktischen Kompetenzen („TOP 10 Anästhesie“). Diese sollten als Mindeststandard nach Absolvierung des PJ-Tertials von allen Studierenden selbstständig beherrscht werden und somit auch prüfbar sein. Dabei folgte man der grundlegenden Frage: *Welche Kompetenzen erwarten wir von approbierten Ärztinnen und Ärzten zum Weiterbildungsbeginn in der Anästhesiologie* (Tab. 1). Der konzeptionelle Aufbau des Curriculums beinhaltet daher als eines der Hauptziele die Durchführung und Prüfung dieser Kompetenzen. Weiterführende Lernziele, angelehnt an die Lernzielkataloge des NKLM und der DGAI, behielten ihre Gültigkeit, wurden jedoch nicht primär geprüft.

**Table 1:** 

Zum Ende des Wahltertials Anästhesiologie kann der/die Studierende im praktischen Jahr als aktives Mitglied des professionellen Behandlungsteams sicher patientennahe klinisch-praktische Kompetenzen situativ adäquat und in einer für die Patientinnen und Patienten respektvollen Weise selbstständig unter Supervision durchführen	
	Der/die Studierende kann …
Anästhesie	*1. Eine Prämedikationsvisite zu einer einfachen Operation bei ASA-I/II-Patienten durchführen* … eine fokussierte, anästhesierelevante körperliche Untersuchung demonstrieren … eine präoperative Gesamtrisikoevaluation durchführen … ein anästhesiologisches Aufklärungsgespräch durchführen
	*2. Ein Basismonitoring zu allen Vitalparametern (Herzfrequenz, Blutdruck, Atmung, Temperatur und S*_*p*_*O*_*2*_*) etablieren und interpretieren*
	*3. Hygienisch einwandfrei und sicher einen peripher-venösen Zugang (≥16* *G) anlegen*
	*4. Die Anästhesieeinleitung bei einem ASA-I/II-Patienten zu einer einfachen Operation durchführen* … Die dosisgerechte Anwendung von Hypnotika, Analgetika und Relaxanzien demonstrieren … Eine Masken-Beutel-Beatmung suffizient durchführen … Den Einsatz eines Guedel- und Wendl-Tubus demonstrieren … Die Atemwegssicherung mit einer Larynxmaske, einem Larynxtubus durchführen
Intensivmedizin	*5. Eine organorientierte, fokussierte klinische Untersuchung am bewusstlosen/sedierten Patienten durchführen und, darauf stützend, die strukturierte Patientenvorstellung/-übergabe demonstrieren*
	*6. Sonographisch folgende anästhesie- und intensivmedizinrelevanten, anatomische Strukturen darstellen: Harnblase, Douglas-Raum, Koller-Pouch, Morison-Pouch, Nieren, 4‑Kammer-Darstellung subkostal des Herzens, inkl. Perikard, Pleura, V. cava inferior, A./V. femoralis, A./V. jugularis interna, A. radialis, A. brachialis*
	*7. Blutkulturen hygienisch einwandfrei abnehmen*
Notfallmedizin	*8. Im Simulator die aktuellen Algorithmen des European Resuscitation Council für den Advanced Life Support des Erwachsenen demonstrieren*
Schmerzmedizin	*9. Eine Schmerzanamnese strukturiert erheben*
	*10. Ein postoperatives Schmerzkonzept erstellen*

Durch die Projektvorstellung und gemeinsame Lernzieldefinition mit der Klinikleitung und der ärztlichen Belegschaft im Rahmen der Lehrkonferenzen konnte der Projektstart im März 2018 mit breiter Zustimmung und Unterstützung aller Entscheidungsträger beginnen. Für die Projektdurchführung, -koordination und -evaluation wurden 2 ärztliche Mitarbeiter von der Klinikleitung berufen, die in einem zeitlichen Umfang von zusammen 8 Arbeitsstunden monatlich für die Projektumsetzung freigestellt wurden.

**Figure 1:**
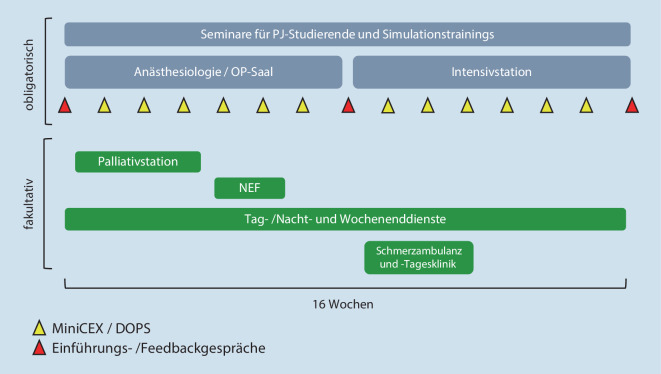

Alle Studierenden wurden obligat jeweils 8 Wochen in der Anästhesiologie und der Intensivmedizin eingesetzt. Fakultativ waren darüber hinaus in dieser Zeit Rotationen in die Palliativ‑, Schmerz- und präklinische Notfallmedizin möglich (Abb. 1). Außerdem wurde bei Interesse ein Einsatz im Dienstsystem ermöglicht (lange Tagdienste, Nacht- und Wochenenddienste). Am Beginn und am Ende jedes Tertials erfolgte ein gemeinsames Einführungs- bzw. Abschlussgespräch aller PJ-Studierenden mit den PJ-Koordinatoren der Klinik und einem Vertreter der Klinikleitung.

Während des Tertials fand 2‑ bis 3‑mal wöchentlich für jeweils eine Stunde ein obligatorisches Seminar für alle PJ-Studierenden der Klinik statt [30]. Hier wurden klinisch-praktische Themen, angelehnt an die „TOP 10 Anästhesie“, lernerzentriert erarbeitet und diskutiert. Das Simulationstraining war an die Seminarreihe zeitlich angelehnt und fand in jedem Tertial für mindestens 3 Stunden statt. Neben dem praktischen Training der ALS-Algorithmen nach den aktuellen Leitlinien des European Resuscitation Council (ERC) wurden hier zusätzlich Aspekte des *Crew Resource Management* (CRM) als Lernziele definiert.

Das Mentoring-Team rekrutierte sich aus ärztlichen Kolleginnen und Kollegen der Klinik mit einer mindestens 2‑jährigen Berufserfahrung in der Anästhesiologie. Um den möglichen Interessentenkreis groß zu halten und die individuelle Motivation in den Vordergrund zu stellen, wurde bei der Auswahl der Mentoren/Mentorinnen auf weitere notwendige Qualifikationen verzichten. Alle Kolleginnen und Kollegen verfügen zu diesem Zeitpunkt der ärztlichen Weiterbildung jedoch schon über Erfahrungen in der studentischen Lehre im Rahmen des Unterrichts am Krankenbett („bedside teaching“ im 4. Studienjahr). Die potenziellen Mentoren/Mentorinnen haben sich bei Interesse jeweils selbstständig bei den Koordinatoren des PJ gemeldet. Jeweils einer Person wurde die Ausbildungsverantwortung für einen/eine Studenten/Studentin übertragen. Vor Aufnahme einer Mentoring-Tätigkeit erfolgte jeweils eine 4‑stündige Schulung. Diese setzte sich aus einer einstündigen allgemeinen Einführung mit der Vermittlung von theoretisch-organisatorischen Inhalten, niedergelegt in einem Mentoring-Manual, sowie einem 3‑stündigen praktischen Feedbacktraining gemeinsam mit PJ-Studierenden und einer Trainerin (Psychologin) der medizinischen Fakultät zusammen. In regelmäßigen Treffen des Mentoring-Teams wurden halbstrukturiert Probleme erfasst und konstruktiv aufgearbeitet.

Die Hauptaufgaben der Mentoren/Mentorinnen umfassten die organisatorische Betreuung sowie die Durchführung der Feedbackgespräche und arbeitsplatzbasierten Prüfungen (Abb. 1). Die Prüfungen wurden formativ mithilfe der standardisierten Prüfungsformate Mini-CEX oder DOPS durchgeführt (Zusatzmaterial online: Supplement 1). Bei beiden Prüfungsformaten handelt es sich um checklistenbasierte Werkzeuge, wobei die entsprechenden Prüfungsbogen und die konzeptionelle Aufarbeitung für die Studierenden zur Lernbegleitung in einem Logbuch niedergelegt und online zugänglich gemacht wurden [30]. Die strukturierten Feedbackgespräche fanden obligatorisch in der Mitte und am Ende des Tertials statt. Als Grundlage hierfür konnten u. a. Einführungsgespräche zwischen Mentor/-in und Mentee am Beginn des Tertials zu Hilfe genommen werden, bei denen Erwartungen und individuelle Ziele erfasst wurden. Darüber hinausgehende Feedbackgespräche konnte selbstständig und bedarfsadaptiert jederzeit integriert werden.

Zur Erstellung von relevanten Items für die Evaluation wurde ein halbstrukturiertes Interview mit der ersten Kohorte zum Tertialende durchgeführt. Hierauf basierend entstand ein Fragebogen als kontinuierliches Evaluationsinstrument mit der quantitativen Erfassung von insgesamt 84 Items und jeweils 4‑ bis 6‑Punkte-Likert-Skalen (4-Punkte: fast nie – sehr häufig, 6‑Punkte: trifft gar nicht zu – trifft voll zu). Zudem erlaubte der Fragebogen qualitative Freitextangaben. Folgende Rubriken wurden evaluiert: allgemeine Angaben, Motivation und Kompetenzerleben, Ablauf und Struktur, PJ-Seminar und Simulation, Mentoring, formative Prüfung und Feedback, Gesamtevaluation. Die Rubrik Motivation und Kompetenzerleben umfasst dabei validierte Items zu folgenden Dimensionen: intrinsische Lernmotivation (5 Items), extrinsische Lernmotivation (3 Items), Kompetenzerleben (4 Items) und Selbstbestimmungserleben (4 Items) [20]. Die einzelnen Teilabschnitte wurden dann jeweils mit einer Gesamtbewertung im Schulnotensystem (Noten 1–6) abschlossen. Der komplette Fragebogen ist als Supplement 2 (Zusatzmaterial online) abrufbar. Die Durchführung und statistische Aufarbeitung der Evaluation erfolgten online mithilfe von EvaSys® (Version 7.1; Fa. Electric Paper Evaluationssystem GmbH, Lüneburg, Deutschland). Die Auswertung der Daten und die statistischen Berechnungen erfolgten darüber hinaus mithilfe von Microsoft Excel® Version 2013 (Fa. Microsoft Corporation, Redmond, WA, USA).

## Ergebnisse

Bis einschließlich September 2019 absolvierten insgesamt 40 Studierende das veränderte Curriculum. Die Rücklaufquote des Fragebogens betrug 80,0 % (*n* = 32). Das Geschlechterverhältnis war ausgeglichen (m = 50,0 %, w = 50,0 %, d = 0 %), und die Mehrzahl der Studierenden absolvierte das Tertial als letzten Abschnitt des PJ (48,4 %). Die fakultative Teilnahme am Dienstsystem außerhalb der Kernarbeitszeiten (81,3 %) wie auch am Notarztdienst war sehr beliebt (87,5 %) und wurde von 96,4 % als sinnvoll empfunden. Rotationen auf die Palliativstation (9,4 %) oder in den Schmerzdienst (28,1 %) wurden hingegen weniger häufig wahrgenommen.

Mit der Organisation des Curriculums war der Großteil der Studierenden zufrieden (Tab. 2). Ablauf und Struktur wurden mit einer Gesamtnote von 1,7 [Median [md] = 2,0/Standardabweichung [sd] = ±0,6] bewertet. Trotz Mentoring wünschte sich knapp ein Drittel der Studierenden mehr Unterstützung zu Beginn des Tertials. Der Wechsel zwischen Arbeitsbereichen verlief für die Mehrheit reibungslos. Nahezu alle Studierenden geben an, dass Ansprechpartner klar definiert waren, bei Problemen zeitnah geholfen und auf persönliche Wünsche eingegangen werden konnte. Die Mehrheit gab an, dass das Tertial sowohl im Bereich der Anästhesie als auch auf der Intensivstation gut strukturiert war.

**Table 2:** 

	*n*	1	2	3	4	5	6	mw	md	sd
Für meinen Einstieg ins Tertial benötige ich mehr Unterstützung	32	31,3	18,8	18,8	21,9	3,1	6,3	2,7	2,5	1,5
Der Wechsel zwischen Operationssaal und Intensivstation verlief reibungslos	32	0	3,1	3,1	12,5	15,6	65,6	5,4	6,0	1,0
Meine Ansprechpartner in der Klinik waren klar definiert	32	0	0	0	12,5	18,8	68,8	5,6	6,0	0,7
Bei Problemen wurde mir zeitnah geholfen	32	0	0	3,1	6,3	28,1	62,5	5,5	6,0	0,8
Auf meine persönlichen Wünsche wurde eingegangen	32	0	0	0	12,5	18,8	68,8	5,6	6,0	0,7
Im Operationssaal war das Tertial gut strukturiert	32	3,1	18,8	12,5	15,6	40,6	9,4	4,0	4,5	1,4
Auf der Intensivstation war das Tertial gut strukturiert	32	0	12,5	0	18,8	37,5	31,3	4,8	5,0	1,3

Darstellung der Werte von „trifft gar nicht zu“ ≙ 1 bis zu „trifft voll zu“ ≙ 6 (Angaben jeweils in %)

*mw* Mittelwert, *md* Median, *sd* Standardabweichung

**Figure 2:**
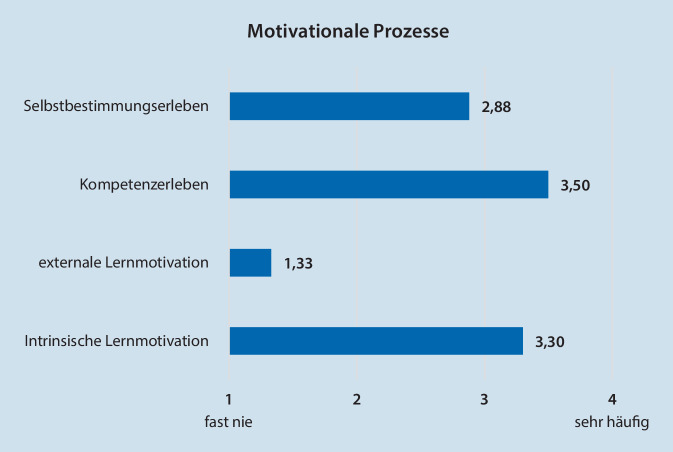

Das Mentoring-basierte Curriculum förderte einen hohen Grad nicht nur intrinsischer Lernmotivation, sondern damit einhergehend auch von Kompetenzerleben und Autonomieempfinden (Abb. 2).

**Figure 3:**
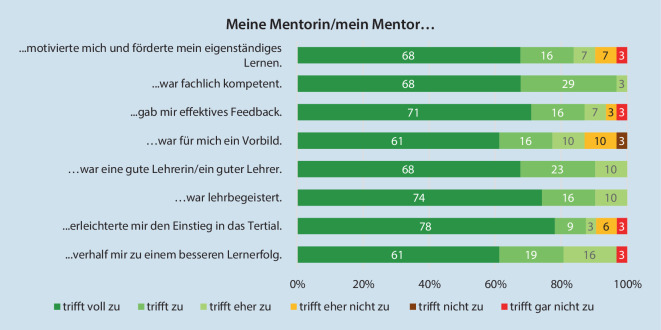

Das Mentoring erleichterte fast allen Studierenden den Einstieg in das Tertial, und alle erlebten das geschulte Mentoring-Team als lehrbegeistert, fachlich kompetent und als gute Ausbildende. Die Mehrheit gab an, dass die Mentorin bzw. der Mentor eine Vorbildfunktion einnahm (87,1 %), effektives Feedback gab (93,6 %), die Studierenden motivierte und ihr eigenständiges Lernen förderte. Fast alle Studierenden (96,8 %) gaben an, dass das Mentoring-Programm ihnen zu einem besseren Lernerfolg verhalf (jeweils Abb. 3). Dennoch wünschen sich im Nachhinein mehr als die Hälfte eine noch engmaschigere Betreuung. Der jeweilige Ausbildungsstand der Mentoren/Mentorinnen war dabei für mehr als 50 % der Befragten von Bedeutung (Tab. 3). Die Gesamtbewertung für das Mentoring erfolgte mit 1,5 [md = 1,0/sd = ±0,8].

**Table 3:** 

	*n*	1	2	3	4	5	6	mw	md	sd
*Mentoring*										
Im Nachhinein würde ich mir eine engmaschigere Betreuung wünschen	32	9,4	25,0	12,5	25,0	9,4	18,8	3,6	4,0	1,6
Der Ausbildungsstand meiner Mentorin/meines Mentors ist mir wichtig	32	18,8	15,6	9,4	25,0	12,5	18,8	3,5	4,0	1,8
*Formative Prüfung und Feedback*										
Arbeitsplatzbasierte Prüfungen sind mir neu	31	22,6	6,5	6,5	6,5	12,9	45,2	4,2	5,0	2,1
Arbeitsplatzbasierte Prüfungen sind hilfreiche Werkzeuge um Kompetenzen zu prüfen	30	0	6,7	16,7	10,0	26,7	40,0	4,8	5,0	1,3
Mini-CEX und DOPS sind effektive Hilfsmittel für den Kompetenzerwerb	30	3,3	6,7	26,7	13,3	26,7	23,3	4,2	4,5	1,4
Ich habe Mini-CEX und DOPS regelmäßig durchgeführt	32	40,6	21,9	9,4	15,6	6,3	6,3	2,4	2,0	1,6
Strukturiertes Feedback war eine neue Erfahrung für mich	28	25,0	14,3	0	7,1	35,7	17,9	3,7	5,0	2,0
Strukturiertes Feedback half mir, effektiv besser zu werden	27	0	3,7	11,1	14,8	22,2	48,1	5,0	5,0	1,2
Strukturiertes Feedback half mir, meine Fähig‑/Fertigkeiten besser einzuschätzen	27	0	3,7	7,4	3,7	37,0	48,1	5,2	5,0	1,1
Strukturiertes Feedback empfand ich als unangenehm	27	63,0	22,2	3,7	3,7	7,4	0	1,7	1,0	1,2
Strukturiertes Feedback empfand ich als unnötig	26	80,8	11,5	7,7	0	0	0	1,3	1,0	0,6
Strukturiertes Feedback empfand ich als ehrlich	27	0	0	3,7	7,4	25,9	63,0	5,5	6,0	0,8
Ich hätte mir mehr strukturiertes Feedback gewünscht	28	17,2	17,2	13,8	10,3	24,1	17,2	3,6	4,0	1,8
*PJ-Seminar und Simulation*										
Die Seminarreihe war praxisbezogen und klinikalltagsrelevant	32	0	0	3,1	9,4	46,9	40,6	5,3	5,0	0,8
Die Seminare waren thematisch sinnvoll gewählt	32	0	3,1	0	3,1	37,5	56,3	5,4	6,0	0,8
Die Seminare waren sinnvoll aufeinander abgestimmt	32	0	6,3	3,1	28,1	43,8	18,8	4,7	5,0	1,0
Die Seminarreihe würde ich weiterempfehlen	32	0	0	3,1	6,3	18,8	71,9	5,6	6,0	0,8
Die Seminare haben mein Verständnis und Lernen gefördert	31	0	0	0	6,5	41,9	51,6	5,5	6,0	0,6
Nach dem Simulationstraining fühlte ich mich auf eine Notfallsituation besser vorbereitet	16	37,5	0	12,5	12,5	12,5	25,0	3,4	3,5	2,1
Das Simulationstraining sollte häufiger angeboten werden	21	9,5	0	9,5	4,8	9,5	66,7	5,0	6,0	1,7
*Gesamtevaluation*										
Ich bin mit meinem Lernerfolg zufrieden	32	0	0	9,4	34,4	34,4	21,9	4,7	5,0	0,9
Das Lernklima empfand ich als angenehm	32	0	0	3,1	15,6	34,4	46,9	5,3	5,0	0,8
Ich habe mich respektiert und wohl gefühlt	31	0	0	9,7	9,7	45,2	35,5	5,1	5,0	0,8
Auf den Teilbereich Anästhesiologie des mündlichen Examens (M3) fühle ich mich gut vorbereitet	32	3,1	3,1	12,5	15,6	53,1	12,5	4,5	5,0	1,2
Bei einem Berufseinstieg als Anästhesist/-in würde ich mich gut vorbereitet fühlen	31	3,2	16,1	9,7	19,4	29,0	22,6	4,2	5,0	1,5
Mein Tertial in der Anästhesiologie und Intensivtherapie würde ich weiterempfehlen	32	0	0	6,3	9,4	12,5	71,9	5,5	6,0	0,9
Interessierten würde ich den Berufsstart an der hiesigen Klinik empfehlen	32	0	0	6,3	18,8	40,6	34,4	5,0	5,0	0,9
Ich könnte mir vorstellen, mich an der hiesigen Klinik zu bewerben	32	6,3	15,6	3,1	9,4	18,8	46,9	4,6	5,0	1,7

*n* = Zahl der Befragungsteilnehmer

Darstellung der Werte von „trifft gar nicht zu“ ≙ 1 bis zu „trifft voll zu“ ≙ 6 (Angaben jeweils in %)

*mw* Mittelwert, *md* Median, *sd* Standardabweichung

Arbeitsplatzbasierte Prüfungen (Mini-CEX, DOPS) waren für fast zwei Drittel der Studierenden neu und damit unbekannt. Gleichzeitig empfanden die Studierenden mit einer hohen Zustimmung die Prüfungen als hilfreiche Werkzeuge, um Kompetenzen zu prüfen, und als ein effektives Hilfsmittel für den Kompetenzerwerb per se. So haben 28,2 % bereits regelmäßig Mini-CEX oder DOPS während des Tertials durchgeführt (≥10/Tertial). Das strukturierte Feedback der Mentorin bzw. des Mentors war für gut 60 % der Studierenden eine neue Erfahrung. Die Studierenden gaben an, dass strukturiertes Feedback ihnen zum einen half, effektiv besser zu werden, und zum anderen, v. a. dazu diente, ihre Fähig- und Fertigkeiten besser einschätzen zu können. Nur 11,1 % empfanden Feedback als unangenehm, keiner als unnötig und die Mehrheit mit 96,3 % als ehrlich. Trotz fest etablierter Feedbackstrukturen wünschte sich im Nachhinein knapp die Hälfte mehr strukturiertes Feedback. Die Rubrik formative Prüfung und Feedback wurde mit einer Gesamtnote von 2,1 bewertet [md = 2,0/sd = ±1,0].

Knapp 97 % der Studierenden beurteilten die Seminarreihe jeweils als praxisbezogen, klinikalltagsrelevant, sinnvoll aufeinander abgestimmt und würden diese anderen Studierenden weiterempfehlen. Fast alle gaben an, dass die Themen sinnvoll gewählt waren und die Seminare Verständnis und Lernen förderten. Der Aussage „Die Referentinnen und Referenten hatten Spaß am Lehren.“ stimmten 37,5 % voll zu, 50,0 % zu und 12,5 % eher zu [md = 5,0/sd = ±0,7]. Nach dem Simulationstraining fühlte sich die Hälfte der Studierenden besser auf eine reale Notfallsituation vorbereitet, und 81 % wünschten sich ein häufigeres Simulationsangebot.

Das Curriculum wurde durch die Studierenden mit der Gesamtnote 1,7 bewertet [md = 2,0/sd = ±0,7]. Gut 90 % der Studierenden waren mit ihrem Lernerfolg zufrieden, und nahezu alle empfanden das Lernklima als angenehm. Der überwiegende Teil der Studierenden hat sich während des Tertials respektiert und wohl gefühlt. Nach Absolvierung des PJ-Curriculums fühlten sich 81,3 % auf den Teilbereich Anästhesiologie innerhalb der abschließenden M3-Staatsexamensprüfung gut vorbereitet, und 71,0 % gaben an, dass sie sich ebenso für einen Weiterbildungsbeginn in der Anästhesiologie gut vorbereitet fühlen. Fast alle würden das PJ in der Klinik und Poliklinik für Anästhesiologie und Intensivtherapie weiterempfehlen. Ebenso viele würden den Weiterbildungsbeginn in der Klinik empfehlen. Gut drei Viertel der PJ-Studierenden konnten sich darüber hinaus vorstellen, nach Erhalt der Approbation selbst eine Bewerbung an der Klinik einzureichen. Der komplette Ergebnisbericht der Evaluation ist als Supplement 3 (Zusatzmaterial online) abrufbar.

## Diskussion

Um das Lernverhalten positiv zu unterstützen und das Lern-Outcome zu verbessern, gilt es, eine übergeordnete Struktur zu etablieren, die die intrinsische Lernmotivation fördert. Das vorgestellte Curriculum ermöglicht durch fest definierte Kompetenzen als Lernzielerwartung, fakultative Arbeitsbereichsrotationen, die Teilnahme am Dienstsystem und das Mentoring-Konzept einen hohen Grad an eigenverantwortlicher Lernsteuerung und fokussierter Lernbegleitung der Studierenden (Kompetenz- und Selbstbestimmungserleben, soziale Eingebundenheit). Dies mündet in einem hohen Maß an intrinsischer Lernmotivation und spiegelt sich u. a. in der hohen Teilnahme am Dienstsystem und Notarztdienst wider [6].

Arbeitsplatzbasierte Prüfungen wurden als formatives Prüfungswerkzeug von den Studierenden als ebenso sinnvolles wie auch als hilfreiches Werkzeug, um Kompetenzen zu prüfen und zu sichern, erlebt. Als mehrheitlich unbekanntes Konzept half es dem überwiegenden Teil der Studierenden, effektiv besser zu werden, v. a., da sich die Studierenden in ihren Fähig- und Fertigkeiten besser einschätzen konnten. Als neu etabliertes Prüfungskonzept führte bereits knapp ein Drittel der Studierenden Mini-CEX oder DOPS regelmäßig durch. Als regelmäßige Durchführung wurde hierbei eine Zahl von mehr als 10 Prüfungen je Tertial gewertet. Diese Anzahl wird auch von der bestehenden Literatur berichtet und zeigt, dass der Erfolg von neu etablierten Konzepten in Organisationen komplex und zeitabhängig ist [18]. Studierende forderten Mini-CEX und DOPS nur im geringen Maße ein, und in einer zeitintensiven Arbeitsumgebung scheinen kurze, halbstrukturierte Feedbackmomente u. U. ausreichend wertvoll zu sein. Den Wunsch nach regelmäßigem Feedback bestätigt auch unsere Analyse: Trotz fest verankerter Feedbackgelegenheiten zu 10 bedarfsorientierten klinisch-praktischen Kompetenzen wünschten sich mehr als die Hälfte im Nachhinein mehr Feedback. Mehr Feedback führt zu besserem Lern-Outcome bei praktischen Kompetenzen, jedoch bleibt weiterhin unklar, wie viel Feedback ein adäquates Nutzen-Ressourcen-Verhältnis aufweist [2].

Die Implementierung neuer Lehrkonzepte ist innerhalb großer Kliniken durch ein von der Klinikleitung unterstütztes, motiviertes Projektteam mit zeitlichen Kontingenten sowie ein geschultes Mentoring-Team zu ermöglichen. Sie wirken als Projektmultiplikatoren und gewährleisten dessen Durchführung. Die Übertragung von Ausbildungsverantwortung an lehrinteressierte Mitarbeiterinnen und Mitarbeitern fördert und unterstützt in ihrer Vorbildrolle nicht nur den Erwerb von Kompetenzen und damit den Lernerfolg der Studierenden, vielmehr kann diese Aufgabe zusätzliche positive Effekte auf die Sozialkompetenz, Motivation und Stressverarbeitung der Studierenden haben [7, 17]. Die Mentorinnen und Mentoren wurden durchweg positiv, insbesondere als motivierend, fachlich kompetent und als effektive Feedbackgebende bewertet. Dennoch wünschte sich knapp die Hälfte im Nachhinein eine engmaschigere Betreuung. Dies verdeutlicht den hohen Stellenwert, der einer differenzierten individuellen Lernbegleitung anzurechnen ist. Eben durch diese unterstützt das Mentoring zusätzlich Coping-Strategien der Studierenden und deren Resilienz, um dem hohen Risiko stressassoziierter Erkrankungen bei Studierenden vorzubeugen [4, 9, 14].

Eine anhand von arbeitsplatzbasierten Prüfungen gelebte Feedbackkultur zwischen Mentor/-in und Mentee kann positive Effekte auf die gesamtklinische Feedback- und Weiterbildungskultur haben. Die Prüfungen fördern implizit Reflexionsfähigkeit, klinische, kommunikative und lehrbezogene Kompetenzen der Mentorinnen und Mentoren selbst und können damit als effektiver Bestandteil der Mitarbeiterentwicklung angesehen werden [21, 23, 26]. Eine strukturierte, bedarfsorientierte Studierendenausbildung im PJ kann zum einen bei Anstellung und Übernahme einen Teil der Einarbeitungsphase abbilden und somit kostenreduzierende Effekte durch Verkürzung der Einarbeitungszeit aufweisen. Zum anderen kann ein Mentoring-System bereits im Vorfeld von der Klinikleitung für eine strukturierte Bewerberevaluation herangezogen werden.

Aufgrund der positiven Evaluationsergebnisse werden wir am aktuellen Mentoring-basierten Curriculum festhalten und dieses konsequent weiterentwickeln. Bestärkt werden wir dabei einerseits durch die durchweg positiven persönlichen Rückmeldungen in den Abschlussgesprächen mit den PJ-Studierenden, aber auch durch die geplanten Änderungen in der ÄApprO [1]. Dem *Masterplan Medizinstudium 2020* folgend, werden in Zukunft im abschließenden Staatsexamen fachspezifische, praktische Kompetenzen standardisiert geprüft [15, 28]. Ziel muss es daher sein, die Studierenden schon früh und regelmäßig mit den neuen arbeitsplatzbasierten Prüfungsformen vertraut zu machen. Das PJ bietet viele Chancen, diese und andere innovativen Konzepte zu erproben und die spätere Implementierung in andere Teile des Medizinstudiums nach Inkrafttreten der anstehenden Änderungen der ÄApprO zu vereinfachen. Ganz im Sinne des Kern-Zyklus haben wir, basierend auf den Ergebnissen der Befragung, erste Anpassungen am Curriculum vorgenommen. Diese sollen dazu führen, dass die Betreuung der Studierenden noch engmaschiger erfolgt und die arbeitsplatzbasierten Prüfungen und somit das strukturierte Feedback noch häufiger durchgeführt werden. Hierzu wurden die Mentoren/Mentorinnen noch einmal gesondert informiert und die Zahl der erhobenen arbeitsplatzbasierten Prüfungen sowohl aufseiten der Mentees als auch der Mentoren/Mentorinnen während eines Tertials durch die Koordinatoren des PJ an der Klinik wiederholt abgefragt. Die folgenden Evaluationen werden zeigen, inwieweit diese Maßnahmen zu einer weiteren Verbesserung beitragen können. Hierbei ist jedoch schon jetzt klar, dass neben der Weiterentwicklung des Curriculums selbst gerade die strukturierte Durchführung arbeitsplatzbasierter Prüfungen mit entsprechendem Feedback personelle und zeitliche Ressourcen bindet. Wir sind jedoch überzeugt, dass es sich hierbei um eine lohnende Investition in den ärztlichen Nachwuchs und die Weiterentwicklung der Klinik handelt.

### Limitationen

Die Bewertung des Tertials mithilfe einer Onlineevaluation bringt per se einige Limitationen mit sich. Allen voran ist sicherlich die subjektive Bewertung der eigenen Kompetenzen durch die Studierenden. Ob diese nun tatsächlich besser in der Ausübung klinisch-praktischen Kompetenzen sind, kann abschließend nicht sicher geklärt werden. Um dies zu klären, wären ein anderes Studiendesign und eine objektive Bewertung der Studierenden notwendig. Künftig kann dies ggf. durch eine Auswertung der geprüften praktischen Kompetenzen im überarbeiteten abschließenden mündlich-praktischen Staatsexamen des Medizinstudiums erfolgen. Die Evaluation stellt jedoch stets den ersten Schritt nach der Implementierung eines neuen Curriculums dar und liefert gerade auch durch ihre Subjektivität wichtige Informationen in Bezug auf Aspekte wie Zufriedenheit und Weiterempfehlungsquote.

Die Rücklaufquote der Befragung lag trotz 2‑maliger E‑Mail-Erinnerung leider nur bei 80 %, sodass nicht die Meinung aller Teilnehmer/-innen des Curriculums einbezogen werden konnte. Über die Gründe der Nichtteilnahme kann nur spekuliert werden. Denkbar wäre v. a. die Überschneidung des Befragungszeitraumes mit der Vorbereitung auf das abschließende Staatsexamen, andere parallel stattfindende Evaluationen oder aber auch fehlende Motivation, da die Ergebnisse das eigene Studium nicht mehr beeinflussen werden. Im Vergleich zu anderen Onlineevaluationen der Medizinischen Fakultät der Universität Leipzig ist die Rücklaufquote aber insgesamt dennoch als hoch einzuschätzen.

## Fazit für die Praxis

- Die Priorisierung fachspezifischer, grundlegender Kompetenzen ermöglicht die klinik- und bedarfsgerechte Wichtung von Lernzielkatalogen.
- Als strukturiertes Feedbackformat werden arbeitsplatzbasierte Prüfungen von Studierenden als hilfreich und sinnvoll erlebt. Sie können das Erlernen praktischer Kompetenzen unterstützen und eine effektive Feedbackkultur sicherstellen.
- Mentoring motiviert die Studierenden und unterstützt das eigenständige Lernen. Es fördert den Lernerfolg hauptsächlich durch Feedback und eine individuelle Lernbegleitung.
- Durch Verschiebung der strukturierten Einarbeitung vom Weiterbildungsbeginn in das praktische Jahr könnten langfristig kostenreduzierende Effekte entstehen, insbesondere durch die Möglichkeit der Beurteilung der Studierenden vor einer möglichen Anstellung.

## Supplementary Information






