# Supplementary material for: Mentoring and workplace-based assessments for final year medical students: An effective way to increase satisfaction and competence?
Source: Anaesthesist. 2020 Dec 14;70(6):486–96. [Article in German] doi: 10.1007/s00101-020-00902-7 (PMC8189941; doi:10.1007/s00101-020-00902-7)
Supplement: Supplementary file 1 [file 101_2020_902_MOESM1_ESM.pdf]

Universitätsklinikum Leipzig
Medizin ist unsere Berufung.

Direct Observation of Procedural Skills (DOPS)

Klinik und Poliklinik für Anästhesiologie und Intensivtherapie

PJ Erfahrung ☐ 1. Tertial ☐ 2. Tertial ☐ 3. Tertial

Klinisch-praktische Kompetenz: ______________________________
TOP 10

Anzahl an DOPS Prüfungen Prüfling
☐ 0 ☐ 1-4 ☐ 5-9 ☐ >10x

Bitte prüfen Sie folgende Bereiche und markieren Sie entsprechend	**Nicht ausreichend**	**Unsicher**	**Kompetent**	N/B*
1. Vorbereitung (Lagerung, Analgesie, …)	☐	☐	☐	☐
2. Technische Fertigkeit	☐	☐	☐	☐
3. Aseptische Technik entsprechend der Hygienerichtlinien	☐	☐	☐	☐
4. Kennt seine Grenzen und Sucht Hilfe wo erforderlich	☐	☐	☐	☐
5. Nachbereitung	☐	☐	☐	☐
6. Kommunikative Kompetenz	☐	☐	☐	☐
7. Professionalität in Durchführung und Auftreten	☐	☐	☐	☐
8. **Gesamtbeurteilung**	☐	☐	☐	☐

*N/B Hier ankreuzen wenn das Verhalten nicht beobachtet wurde

Bitte benutzen Sie dieses Feld für Stärken und etwaige Verbesserungsvorschläge (Strukturiertes Feedback)

______________________________ ______________________________
Unterschrift Student/-in Datum, Unterschrift Arzt/Ärztin, Stempel

Universitätsklinikum Leipzig
Medizin ist unsere Berufung.

Mini Clinical Evaluation Exercise (Mini-CEX)
Klinik und Poliklinik für Anästhesiologie und Intensivtherapie

PJ Erfahrung ☐ 1. Tertial ☐ 2. Tertial ☐ 3. Tertial

Klinisch-kommunikative Kompetenz: ________________________________
TOP 10

Anzahl an Mini-CEX Prüfungen Prüfling
☐ 0 ☐ 1-4 ☐ 5-9 ☐ >10x

Bitte prüfen Sie folgende Bereiche und markieren Sie entsprechend	**Nicht ausreichend**	**Unsicher**	**Kompetent**	N/B*
1. Anamnese	☐	☐	☐	☐
2. Klinische Untersuchung	☐	☐	☐	☐
3. Kommunikative Kompetenz	☐	☐	☐	☐
4. Klinisches Urteilsvermögen	☐	☐	☐	☐
5. Professionalität in Durchführung und Auftreten	☐	☐	☐	☐
6. Organisation und Effizienz	☐	☐	☐	☐
7. **Gesamtbeurteilung**	☐	☐	☐	☐

*N/B Hier ankreuzen wenn das Verhalten nicht beobachtet wurde

Bitte benutzen Sie dieses Feld für Stärken und etwaige Verbesserungsvorschläge (Strukturiertes Feedback)

_______________________________ Unterschrift Student/-in

_______________________________ Datum, Unterschrift Arzt/Ärztin, Stempel
