# Supplementary material for: Mentoring and workplace-based assessments for final year medical students: An effective way to increase satisfaction and competence?
Source: Anaesthesist. 2020 Dec 14;70(6):486–96. [Article in German] doi: 10.1007/s00101-020-00902-7 (PMC8189941; doi:10.1007/s00101-020-00902-7)
Supplement: Supplementary file 2 [file 101_2020_902_MOESM2_ESM.pdf]

MUSTER

EvaSys	Evaluation Tertial Anästhesiologie und Intensivtherapie an der Universitätsklinik Leipzig	Electric Paper

Universitätsklinikum Leipzig

Klinik und Poliklinik für Anästhesiologie und Version Studierende

Bitte so markieren: ☐ ☒ ☐ ☐ ☐ Bitte verwenden Sie einen Kugelschreiber oder nicht zu starken Filzstift. Dieser Fragebogen wird maschinell erfasst.

Korrektur: ☐ ■ ☐ ☒ ☐ Bitte beachten Sie im Interesse einer optimalen Datenerfassung die links gegebenen Hinweise beim Ausfüllen.

1. Allgemeine Angaben

1.1	Bitte geben Sie Ihr Geschlecht an.	☐ männlich	☐ weiblich	☐ sonstige
1.2	In welchem PJ-Tertial waren Sie an unserer Klinik?	☐ 1. Tertial	☐ 2. Tertial	☐ 3. Tertial
1.3	Haben Sie an Diensten außerhalb der Kernarbeitszeiten (FL, S, SN, N, feiertags) teilgenommen?	☐ Ja	☐ Nein	
1.4	Haben Sie am Notarztdienst (NEF Mitte) teilgenommen?	☐ Ja	☐ Nein	
1.5	Sind Sie auf die Palliativstation rotiert?	☐ Ja	☐ Nein	
1.6	Haben Sie am Schmerzdienst teilgenommen?	☐ Ja	☐ Nein	

MUSTER

2. Motivation und Kompetenzerleben

Während des PJ-Tertials Anästhesiologie und Intensivtherapie...

2.1	...erlebte ich mich als neugierig und wissbegierig.	fast nie ☐	☐	☐	☐ sehr häufig
2.2	...machte mir das Arbeiten Spaß.	fast nie ☐	☐	☐	☐ sehr häufig
2.3	...war ich von der Sache so fasziniert, dass ich alles um mich herum vergaß.	fast nie ☐	☐	☐	☐ sehr häufig
2.4	...fand ich das Lernen richtig spannend.	fast nie ☐	☐	☐	☐ sehr häufig
2.5	...hatte ich das Gefühl, mich kaum von der Sache lösen zu können.	fast nie ☐	☐	☐	☐ sehr häufig
2.6	...hatte ich den Eindruck nur aufgrund des mündlichen Examens (M3) zu lernen.	fast nie ☐	☐	☐	☐ sehr häufig
2.7	...empfand ich das Lernen als anstrengend.	fast nie ☐	☐	☐	☐ sehr häufig
2.8	...hatte ich das Gefühl mich zum Arbeiten zwingen zu müssen.	fast nie ☐	☐	☐	☐ sehr häufig
2.9	...merkte ich selbst, was ich kann bzw. was ich noch nicht kann.	fast nie ☐	☐	☐	☐ sehr häufig
2.10	...fühlte ich mich aktiv.	fast nie ☐	☐	☐	☐ sehr häufig
2.11	...ergaben sich aus dem klinischen Alltag Probleme, mit denen ich mich eingehender beschäftigen möchte.	fast nie ☐	☐	☐	☐ sehr häufig
2.12	...hatte ich den Eindruck, meinen Lernerfolg selbst steuern zu können.	fast nie ☐	☐	☐	☐ sehr häufig
2.13	...fühlte ich mich ernst genommen.	fast nie ☐	☐	☐	☐ sehr häufig
2.14	...fühlte ich mich stark kontrolliert.	fast nie ☐	☐	☐	☐ sehr häufig
2.15	...hatte ich das Gefühl, etwas zu tun, was ich auch selber tun wollte.	fast nie ☐	☐	☐	☐ sehr häufig
2.16	...hatte ich das Gefühl, Entscheidungsspielräume zu haben.	fast nie ☐	☐	☐	☐ sehr häufig

3. Ablauf und Struktur

3.1	Für meinen Einstieg ins Tertial benötige ich mehr Unterstützung.	trifft gar nicht zu ☐	☐	☐	☐	☐	☐ trifft voll zu
3.2	Die Teilnahme an Diensten empfand ich als sinnvoll.	trifft gar nicht zu ☐	☐	☐	☐	☐	☐ trifft voll zu
3.3	Im Operationssaal war das Tertial gut strukturiert.	trifft gar nicht zu ☐	☐	☐	☐	☐	☐ trifft voll zu
3.4	Im Operationssaal war das Tertial fachlich anspruchsvoll.	trifft gar nicht zu ☐	☐	☐	☐	☐	☐ trifft voll zu
3.5	Auf der Intensivstation war das Tertial gut strukturiert.	trifft gar nicht zu ☐	☐	☐	☐	☐	☐ trifft voll zu
3.6	Auf der Intensivstation war das Tertial fachlich anspruchsvoll.	trifft gar nicht zu ☐	☐	☐	☐	☐	☐ trifft voll zu
3.7	Das Tertial bot mir ausreichend Zeit für Selbststudium.	trifft gar nicht zu ☐	☐	☐	☐	☐	☐ trifft voll zu
3.8	Meine Ansprechpartner in der Klinik waren klar definiert.	trifft gar nicht zu ☐	☐	☐	☐	☐	☐ trifft voll zu
3.9	Die Organisation von Hospitationstagen (Palliativmedizin, Notfallmedizin, Schmerzmedizin) war einfach.	trifft gar nicht zu ☐	☐	☐	☐	☐	☐ trifft voll zu
3.10	Der Wechsel zwischen Operationssaal und Intensivstation verlief reibungslos.	trifft gar nicht zu ☐	☐	☐	☐	☐	☐ trifft voll zu
3.11	Auf meine persönlichen Wünsche wurde eingegangen.	trifft gar nicht zu ☐	☐	☐	☐	☐	☐ trifft voll zu
3.12	Bei Problemen wurde mir zeitnah geholfen.	trifft gar nicht zu ☐	☐	☐	☐	☐	☐ trifft voll zu
3.13	Ich fühlte mich als Teil des Behandlungteams.	trifft gar nicht zu ☐	☐	☐	☐	☐	☐ trifft voll zu

3.14	**Gesamtnote *Ablauf und Struktur* (Schulnoten)**	1 ☐	☐	☐	☐	☐	☐ 6

Bitte beschreiben Sie Probleme, die Sie erlebt haben so präzise wie möglich oder loben Sie was Sie als besonders positiv empfanden.

3.15 **Feedback und Kommentare zu *Ablauf und Struktur***

4. PJ-Seminar und Simulation

Die Seminarreihe für PJ-Studierende...

4.1	...habe ich immer besucht.	trifft gar nicht zu	☐	☐	☐	☐	☐	☐ trifft voll zu
4.2	...war praxisbezogen und klinikalltagsrelevant.	trifft gar nicht zu	☐	☐	☐	☐	☐	☐ trifft voll zu
4.3	...würde ich weiterempfehlen.	trifft gar nicht zu	☐	☐	☐	☐	☐	☐ trifft voll zu

Die Seminare...

4.4	...waren thematisch sinnvoll gewählt.	trifft gar nicht zu	☐	☐	☐	☐	☐	☐ trifft voll zu
4.5	...waren sinnvoll aufeinander abgestimmt.	trifft gar nicht zu	☐	☐	☐	☐	☐	☐ trifft voll zu
4.6	...haben mein Verständnis und Lernen gefördert.	trifft gar nicht zu	☐	☐	☐	☐	☐	☐ trifft voll zu
4.7	...fanden regelmäßig statt.	trifft gar nicht zu	☐	☐	☐	☐	☐	☐ trifft voll zu
4.8	Die Referntinnen und Referenten hatten Spaß am Lehren.	trifft gar nicht zu	☐	☐	☐	☐	☐	☐ trifft voll zu

Das Simulationstraining...

4.9	...war gut strukturiert.	trifft gar nicht zu	☐	☐	☐	☐	☐	☐ trifft voll zu
4.10	...hatte einen persönlichen Mehrwert für mich.	trifft gar nicht zu	☐	☐	☐	☐	☐	☐ trifft voll zu
4.11	...sollte häufiger angeboten werden.	trifft gar nicht zu	☐	☐	☐	☐	☐	☐ trifft voll zu
4.12	Nach dem Simulationstraining fühlte ich mich auf eine Notfallsituation besser vorbereitet.	trifft gar nicht zu	☐	☐	☐	☐	☐	☐ trifft voll zu

---

4.13	**Gesamtnote *PJ-Seminar und Simulationstraining* (Schulnoten)**	1	☐	☐	☐	☐	☐	☐ 6

Bitte beschreiben Sie Probleme, die Sie erlebt haben so präzise wie möglich oder loben Sie was Sie als besonders positiv empfanden.

4.14 **Feedback und Kommentare zu *PJ-Seminar und Simulationstraining***

MUSTER

5. Mentoring

	**Meine Mentorin/mein Mentor...**								
5.1	...erleichterte mir den Einstieg in das Tertial.	trifft gar nicht zu	☐	☐	☐	☐	☐	☐	trifft voll zu
5.2	...ist lehrbegeistert.	trifft gar nicht zu	☐	☐	☐	☐	☐	☐	trifft voll zu
5.3	...ist eine gute Lehrerin/ein guter Lehrer.	trifft gar nicht zu	☐	☐	☐	☐	☐	☐	trifft voll zu
5.4	...ist für mich ein Vorbild.	trifft gar nicht zu	☐	☐	☐	☐	☐	☐	trifft voll zu
5.5	...gab mir effektives Feedback.	trifft gar nicht zu	☐	☐	☐	☐	☐	☐	trifft voll zu
5.6	...war fachlich kompetent.	trifft gar nicht zu	☐	☐	☐	☐	☐	☐	trifft voll zu
5.7	...motivierte mich und förderte mein eigenständiges Lernen.	trifft gar nicht zu	☐	☐	☐	☐	☐	☐	trifft voll zu
5.8	Das Mentoring-Programm verhalf mir zu einem besseren Lernerfolg.	trifft gar nicht zu	☐	☐	☐	☐	☐	☐	trifft voll zu
5.9	Der Ausbildungsstand (Arzt in Weiterbildung, Facharzt) meiner Mentorin/meines Mentors ist mir wichtig.	trifft gar nicht zu	☐	☐	☐	☐	☐	☐	trifft voll zu
5.10	Ich empfinde die Kontaktaufnahme zum Tertialbeginn durch das Mentoring-Team als unnötig.	trifft gar nicht zu	☐	☐	☐	☐	☐	☐	trifft voll zu
5.11	Ich fühlte mich durch meine Mentorin/mein Mentor gut begleitet.	trifft gar nicht zu	☐	☐	☐	☐	☐	☐	trifft voll zu
5.12	Im Nachhinein würde ich mir eine engmaschigere Betreuung wünschen.	trifft gar nicht zu	☐	☐	☐	☐	☐	☐	trifft voll zu

5.13 **Gesamtnote zu *Mentoring* (Schulnoten)** 1 ☐ ☐ ☐ ☐ ☐ ☐ 6

Bitte beschreiben Sie Probleme, die Sie erlebt haben so präzise wie möglich oder loben Sie was Sie als besonders positiv empfanden.

5.14 **Feedback und Kommentare zum *Mentoring***

MUSTER

MUSTER

6. Formative Prüfung und Feedback

Arbeitsplatz-basierte Prüfungen, wie z.B. Direct Observation of Procedural Skills (DOPS) oder Mini Clinical Evaluation Exercise (Mini-CEX), sind formative Assessement-Tools (d.h. keine Benotung), die hauptsächlich durch das fest integrierte, strukturierte Feedback leben und wirken. Feedback gehört nachweislich zu den effektivsten Methoden um eine Verhaltensänderung herbei zu führen. Somit ist es weniger eine Überprüfung, sondern vielmehr eine Lehrmethode. Mini-CEX sind für die Evaluation kommunikativer und klinisch-praktischer Kompetenzen ausgelegt. DOPS hingegen eignet sich sehr gut um praktische Fertigkeiten und Interventionen zu beurteilen.

6.1	Arbeitsplatz-basierte Prüfungen sind mir neu.	trifft gar nicht zu	☐	☐	☐	☐	☐	☐	trifft voll zu
6.2	Arbeitsplatz-basierte Prüfungen sind hilfreiche Werkzeuge um Kompetenzen zu prüfen.	trifft gar nicht zu	☐	☐	☐	☐	☐	☐	trifft voll zu
6.3	Ich habe Mini-CEX und DOPS regelmäßig durchgeführt.	trifft gar nicht zu	☐	☐	☐	☐	☐	☐	trifft voll zu
6.4	Mini-CEX und DOPS sind effektive Hilfsmittel für den Kompetenzerwerb.	trifft gar nicht zu	☐	☐	☐	☐	☐	☐	trifft voll zu
6.5	Das strukturierte Feedback meiner Mentorin/meines Mentors empfand ich als hilfreich.	trifft gar nicht zu	☐	☐	☐	☐	☐	☐	trifft voll zu

Strukturiertes Feedback während des Tertials...

6.6	...war eine neue Erfahrung für mich.	trifft gar nicht zu	☐	☐	☐	☐	☐	☐	trifft voll zu
6.7	...half mir effektiv besser zu werden.	trifft gar nicht zu	☐	☐	☐	☐	☐	☐	trifft voll zu
6.8	...half mir meine Fähig- und Fertigkeiten besser einzuschätzen.	trifft gar nicht zu	☐	☐	☐	☐	☐	☐	trifft voll zu
6.9	...empfand ich als unangenehm.	trifft gar nicht zu	☐	☐	☐	☐	☐	☐	trifft voll zu
6.10	...empfand ich als ehrlich.	trifft gar nicht zu	☐	☐	☐	☐	☐	☐	trifft voll zu
6.11	...war unnötig.	trifft gar nicht zu	☐	☐	☐	☐	☐	☐	trifft voll zu
6.12	Ich hätte mir mehr strukturiertes Feedback gewünscht.	trifft gar nicht zu	☐	☐	☐	☐	☐	☐	trifft voll zu

6.13	**Gesamtnote zu *Formative Prüfung und Feedback* (Schulnoten)**	1	☐	☐	☐	☐	☐	☐	6

Bitte beschreiben Sie Probleme, die Sie erlebt haben so präzise wie möglich oder loben Sie was Sie als besonders positiv empfanden.

6.14 **Feedback und Kommentare zu *Formative Prüfung und Feedback***

MUSTER

7. Gesamtevaluation

7.1	Ich bin mit meinem Lernerfolg zufrieden.	trifft gar nicht zu	☐	☐	☐	☐	☐	☐	trifft voll zu
7.2	Das Lernklima empfand ich als angenehm.	trifft gar nicht zu	☐	☐	☐	☐	☐	☐	trifft voll zu
7.3	Ich habe mich respektiert und wohl gefühlt.	trifft gar nicht zu	☐	☐	☐	☐	☐	☐	trifft voll zu
7.4	Auf den Teilbereich Anästhesiologie des mündlichen Examens (M3) fühle ich mich nach dem Tertial gut vorbereitet.	trifft gar nicht zu	☐	☐	☐	☐	☐	☐	trifft voll zu
7.5	Bei einem Berufseinstieg als Anästhesistin/Anästhesist würde ich mich gut vorbereitet fühlen.	trifft gar nicht zu	☐	☐	☐	☐	☐	☐	trifft voll zu
7.6	Mein Tertial in der Anästhesiologie und Intensivtherapie würde ich weiter empfehlen.	trifft gar nicht zu	☐	☐	☐	☐	☐	☐	trifft voll zu
7.7	Interessierten würde ich den Berufsstart an der hiesigen Klinik und Poliklinik für Anästhesiologie und Intensivtherapie empfehlen.	trifft gar nicht zu	☐	☐	☐	☐	☐	☐	trifft voll zu
7.8	Ich könnte mir vorstellen mich an der hiesigen Klinik für Anästhesiologie und Intensivtherapie zu bewerben.	trifft gar nicht zu	☐	☐	☐	☐	☐	☐	trifft voll zu

7.9 **Gesamtnote des PJ-Tertials (Schulnoten)** 1 ☐ ☐ ☐ ☐ ☐ ☐ 6

Bitte beschreiben Sie Probleme, die Sie erlebt haben so präzise wie möglich oder loben Sie was Sie als besonders positiv empfanden.

7.10 **Feedback und Kommentare im Allgemeinen**

MUSTER
