# Supplementary material for: Mentoring and workplace-based assessments for final year medical students: An effective way to increase satisfaction and competence?
Source: Anaesthesist. 2020 Dec 14;70(6):486–96. [Article in German] doi: 10.1007/s00101-020-00902-7 (PMC8189941; doi:10.1007/s00101-020-00902-7)
Supplement: Supplementary file 3 [file 101_2020_902_MOESM3_ESM.pdf]

Praktisches Jahr in der KAI

2018 bis 2020
Erfasste Fragebögen = 32

Auswertungsteil der geschlossenen Fragen

Legende

Fragetext

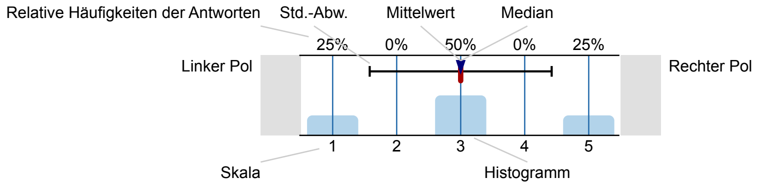

n=Anzahl
mw=Mittelwert
md=Median
s=Std.-Abw.
E.=Enthaltung

1. Allgemeine Angaben

1.1) Bitte geben Sie Ihr Geschlecht an.

n=32

männlich	50%
weiblich	50%
sonstige	0%

1.2) In welchem PJ-Tertial waren Sie an unserer Klinik?

n=31

1. Tertial	29%
2. Tertial	22.6%
3. Tertial	48.4%

1.3) Haben Sie an Diensten außerhalb der Kernarbeitszeiten (FL, S, SN, N, feiertags) teilgenommen?

n=32

Ja	81.3%
Nein	18.8%

1.4) Haben Sie am Notarztdienst (NEF Mitte) teilgenommen?

n=32

Ja	87.5%
Nein	12.5%

1.5) Sind Sie auf die Palliativstation rotiert?

n=32

Ja	9.4%
Nein	90.6%

1.6) Haben Sie am Schmerzdienst teilgenommen?

n=32

Ja	28.1%
Nein	71.9%

2. Motivation und Kompetenzerleben

			1	2	3	4		
2.1)	...erlebte ich mich als neugierig und wissbegierig.	fast nie	0%	0%	43,8%	56,3%	sehr häufig	n=32 mw=3,6 md=4 s=0,5
2.2)	...machte mir das Arbeiten Spaß.	fast nie	0%	6,3%	34,4%	59,4%	sehr häufig	n=32 mw=3,5 md=4 s=0,6
2.3)	...war ich von der Sache so fasziniert, dass ich alles um mich herum vergaß.	fast nie	12,5%	34,4%	37,5%	15,6%	sehr häufig	n=32 mw=2,6 md=3 s=0,9
2.4)	...fand ich das Lernen richtig spannend.	fast nie	0%	12,5%	43,8%	43,8%	sehr häufig	n=32 mw=3,3 md=3 s=0,7
2.5)	...hatte ich das Gefühl, mich kaum von der Sache lösen zu können.	fast nie	12,5%	37,5%	40,6%	9,4%	sehr häufig	n=32 mw=2,5 md=2,5 s=0,8
2.6)	...hatte ich den Eindruck nur aufgrund des mündlichen Examens (M3) zu lernen.	fast nie	81,3%	6,3%	12,5%	0%	sehr häufig	n=32 mw=1,3 md=1 s=0,7
2.7)	...empfand ich das Lernen als anstrengend.	fast nie	31,3%	46,9%	15,6%	6,3%	sehr häufig	n=32 mw=2 md=2 s=0,9
2.8)	...hatte ich das Gefühl mich zum Arbeiten zwingen zu müssen.	fast nie	59,4%	34,4%	6,3%	0%	sehr häufig	n=32 mw=1,5 md=1 s=0,6
2.9)	...merkte ich selbst, was ich kann bzw. was ich noch nicht kann.	fast nie	0%	3,1%	34,4%	62,5%	sehr häufig	n=32 mw=3,6 md=4 s=0,6
2.10)	...fühlte ich mich aktiv.	fast nie	0%	28,1%	34,4%	37,5%	sehr häufig	n=32 mw=3,1 md=3 s=0,8
2.11)	...ergaben sich aus dem klinischen Alltag Probleme, mit denen ich mich eingehender beschäftigen möchte.	fast nie	0%	3,1%	43,8%	53,1%	sehr häufig	n=32 mw=3,5 md=4 s=0,6
2.12)	...hatte ich den Eindruck, meinen Lernerfolg selbst steuern zu können.	fast nie	3,1%	15,6%	62,5%	18,8%	sehr häufig	n=32 mw=3 md=3 s=0,7
2.13)	...fühlte ich mich ernst genommen.	fast nie	0%	15,6%	40,6%	43,8%	sehr häufig	n=32 mw=3,3 md=3 s=0,7

2.14) ...fühlte ich mich stark kontrolliert.

fast nie
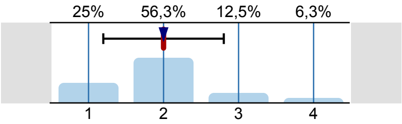

sehr häufig

n=32
mw=2
md=2
s=0,8

2.15) ...hatte ich das Gefühl, etwas zu tun, was ich auch selber tun wollte.

fast nie
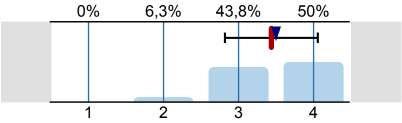

sehr häufig

n=32
mw=3,4
md=3,5
s=0,6

2.16) ...hatte ich das Gefühl, Entscheidungsspielräume zu haben.

fast nie
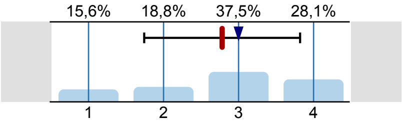

sehr häufig

n=32
mw=2,8
md=3
s=1

3. Ablauf und Struktur

3.1) Für meinen Einstieg ins Tertial benötige ich mehr Unterstützung.

trifft gar nicht zu
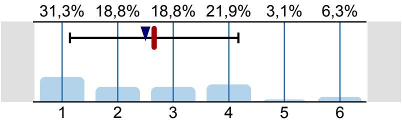

trifft voll zu

n=32
mw=2,7
md=2,5
s=1,5

3.2) Die Teilnahme an Diensten empfand ich als sinnvoll.

trifft gar nicht zu
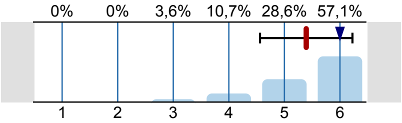

trifft voll zu

n=28
mw=5,4
md=6
s=0,8

3.3) Im Operationssaal war das Tertial gut strukturiert.

trifft gar nicht zu
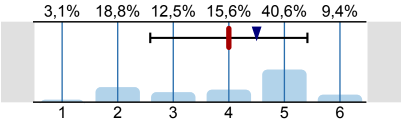

trifft voll zu

n=32
mw=4
md=4,5
s=1,4

3.4) Im Operationssaal war das Tertial fachlich anspruchsvoll.

trifft gar nicht zu
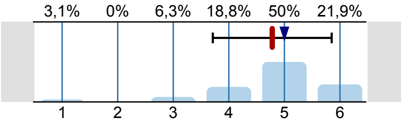

trifft voll zu

n=32
mw=4,8
md=5
s=1,1

3.5) Auf der Intensivstation war das Tertial gut strukturiert.

trifft gar nicht zu
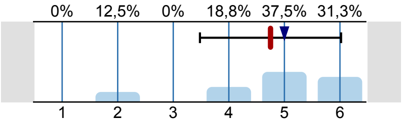

trifft voll zu

n=32
mw=4,8
md=5
s=1,3

3.6) Auf der Intensivstation war das Tertial fachlich anspruchsvoll.

trifft gar nicht zu
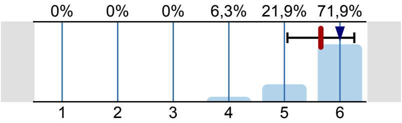

trifft voll zu

n=32
mw=5,7
md=6
s=0,6

3.7) Das Tertial bot mir ausreichend Zeit für Selbststudium.

trifft gar nicht zu
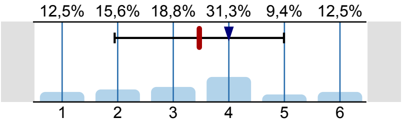

trifft voll zu

n=32
mw=3,5
md=4
s=1,5

3.8) Meine Ansprechpartner in der Klinik waren klar definiert.

trifft gar nicht zu
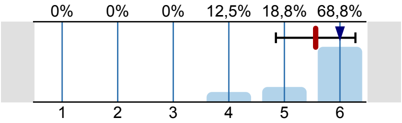

trifft voll zu

n=32
mw=5,6
md=6
s=0,7

3.9) Die Organisation von Hospitationstagen (Palliativmedizin, Notfallmedizin, Schmerzmedizin) war einfach.

trifft gar nicht zu
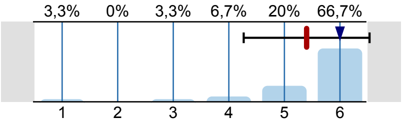

trifft voll zu

n=30
mw=5,4
md=6
s=1,1

3.10) Der Wechsel zwischen Operationssaal und Intensivstation verlief reibungslos.

trifft gar nicht zu
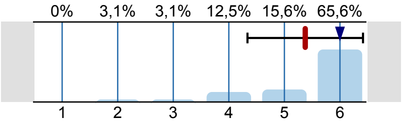

trifft voll zu

n=32
mw=5,4
md=6
s=1

3.11) Auf meine persönlichen Wünsche wurde eingegangen.

trifft gar nicht zu

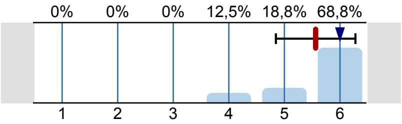

trifft voll zu

n=32
mw=5,6
md=6
s=0,7

3.12) Bei Problemen wurde mir zeitnah geholfen.

trifft gar nicht zu

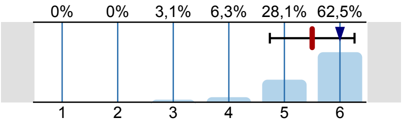

trifft voll zu

n=32
mw=5,5
md=6
s=0,8

3.13) Ich fühlte mich als Teil des Behandlungteams.

trifft gar nicht zu

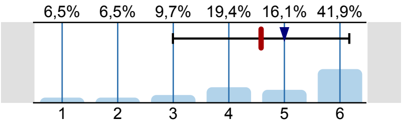

trifft voll zu

n=31
mw=4,6
md=5
s=1,6

3.14) **Gesamtnote *Ablauf und Struktur* (Schulnoten)**

1

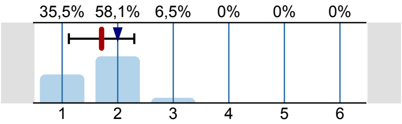

6

n=31
mw=1,7
md=2
s=0,6

4. PJ-Seminar und Simulation

4.1) ...habe ich immer besucht.

trifft gar nicht zu

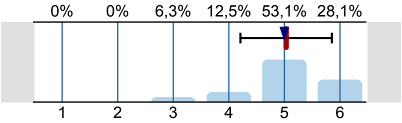

trifft voll zu

n=32
mw=5
md=5
s=0,8

4.2) ...war praxisbezogen und klinikalltagsrelevant.

trifft gar nicht zu

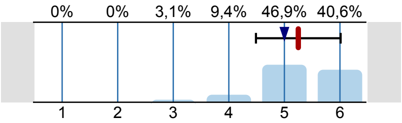

trifft voll zu

n=32
mw=5,3
md=5
s=0,8

4.3) ...würde ich weiterempfehlen.

trifft gar nicht zu

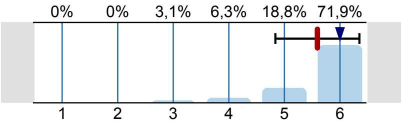

trifft voll zu

n=32
mw=5,6
md=6
s=0,8

4.4) ...waren thematisch sinnvoll gewählt.

trifft gar nicht zu

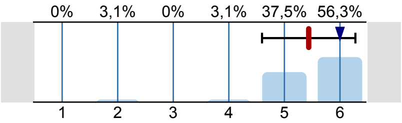

trifft voll zu

n=32
mw=5,4
md=6
s=0,8

4.5) ...waren sinnvoll aufeinander abgestimmt.

trifft gar nicht zu

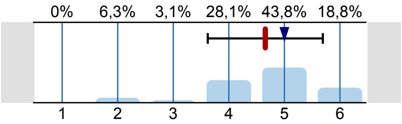

trifft voll zu

n=32
mw=4,7
md=5
s=1

4.6) ...haben mein Verständnis und Lernen gefördert.

trifft gar nicht zu

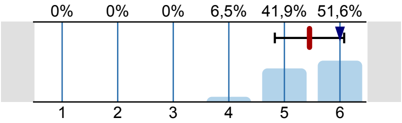

trifft voll zu

n=31
mw=5,5
md=6
s=0,6

4.7) ...fanden regelmäßig statt.

trifft gar nicht zu

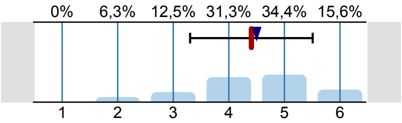

trifft voll zu

n=32
mw=4,4
md=4,5
s=1,1

4.8) Die Referntinnen und Referenten hatten Spaß am Lehren.

trifft gar nicht zu

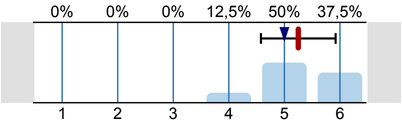

trifft voll zu

n=32
mw=5,3
md=5
s=0,7

4.9) ...war gut strukturiert.

trifft gar nicht zu

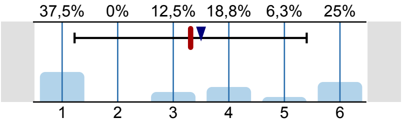

trifft voll zu

n=16
mw=3,3
md=3,5
s=2,1

4.10) ...hatte einen persönlichen Mehrwert für mich.

trifft gar nicht zu	1	2	3	4	5	6	trifft voll zu	n=16, mw=3,9, md=4,5, s=2,2
	31,3%	0%	12,5%	6,3%	6,3%	43,8%		

4.11) ...sollte häufiger angeboten werden.

trifft gar nicht zu	1	2	3	4	5	6	trifft voll zu	n=21, mw=5, md=6, s=1,7
	9,5%	0%	9,5%	4,8%	9,5%	66,7%		

4.12) Nach dem Simulationstraining fühlte ich mich auf eine Notfallsituation besser vorbereitet.

trifft gar nicht zu	1	2	3	4	5	6	trifft voll zu	n=16, mw=3,4, md=3,5, s=2,1
	37,5%	0%	12,5%	12,5%	12,5%	25%		

4.13) **Gesamtnote *PJ-Seminar und Simulationstraining* (Schulnoten)**

1	1	2	3	4	5	6	6	n=31, mw=2,1, md=2, s=0,7
	19,4%	54,8%	25,8%	0%	0%	0%		

5. Mentoring

5.1) ...erleichterte mir den Einstieg in das Tertial.

trifft gar nicht zu	1	2	3	4	5	6	trifft voll zu	n=32, mw=5,5, md=6, s=1,2
	3,1%	0%	6,3%	3,1%	9,4%	78,1%		

5.2) ...ist lehrbegeistert.

trifft gar nicht zu	1	2	3	4	5	6	trifft voll zu	n=31, mw=5,6, md=6, s=0,7
	0%	0%	0%	9,7%	16,1%	74,2%		

5.3) ...ist eine gute Lehrerin/ein guter Lehrer.

trifft gar nicht zu	1	2	3	4	5	6	trifft voll zu	n=31, mw=5,6, md=6, s=0,7
	0%	0%	0%	9,7%	22,6%	67,7%		

5.4) ...ist für mich ein Vorbild.

trifft gar nicht zu	1	2	3	4	5	6	trifft voll zu	n=31, mw=5,2, md=6, s=1,2
	0%	3,2%	9,7%	9,7%	16,1%	61,3%		

5.5) ...gab mir effektives Feedback.

trifft gar nicht zu	1	2	3	4	5	6	trifft voll zu	n=31, mw=5,5, md=6, s=1,1
	3,2%	0%	3,2%	6,5%	16,1%	71%		

5.6) ...war fachlich kompetent.

trifft gar nicht zu	1	2	3	4	5	6	trifft voll zu	n=31, mw=5,6, md=6, s=0,6
	0%	0%	0%	3,2%	29%	67,7%		

5.7) ...motivierte mich und förderte mein eigenständiges Lernen.

trifft gar nicht zu	1	2	3	4	5	6	trifft voll zu	n=31, mw=5,4, md=6, s=1,2
	3,2%	0%	6,5%	6,5%	16,1%	67,7%		

5.8) Das Mentoring-Programm verhalf mir zu einem besseren Lernerfolg.

trifft gar nicht zu	1	2	3	4	5	6	trifft voll zu	n=31, mw=5,3, md=6, s=1,1
	3,2%	0%	0%	16,1%	19,4%	61,3%		

5.9) Der Ausbildungsstand (Arzt in Weiterbildung, Facharzt) meiner Mentorin/meines Mentors ist mir wichtig.

trifft gar nicht zu	1	2	3	4	5	6	trifft voll zu	n=32, mw=3,5, md=4, s=1,8
	18,8%	15,6%	9,4%	25%	12,5%	18,8%		

5.10) Ich empfinde die Kontaktaufnahme zum Tertialbeginn durch das Mentoring-Team als unnötig.

trifft gar nicht zu 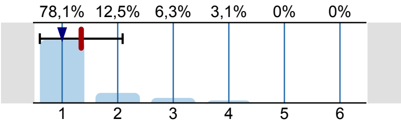

trifft voll zu

n=32
mw=1,3
md=1
s=0,7

5.11) Ich fühlte mich durch meine Mentorin/mein Mentor gut begleitet.

trifft gar nicht zu 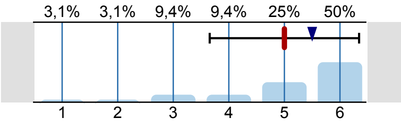

trifft voll zu

n=32
mw=5
md=5,5
s=1,3

5.12) Im Nachhinein würde ich mir eine engmaschigere Betreuung wünschen.

trifft gar nicht zu 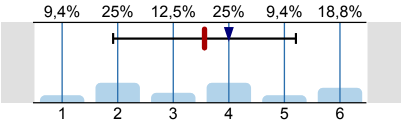

trifft voll zu

n=32
mw=3,6
md=4
s=1,6

5.13) **Gesamtnote zu *Mentoring* (Schulnoten)**

1 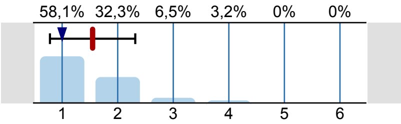

6

n=31
mw=1,5
md=1
s=0,8

6. Formative Prüfung und Feedback

6.1) Arbeitsplatz-basierte Prüfungen sind mir neu.

trifft gar nicht zu 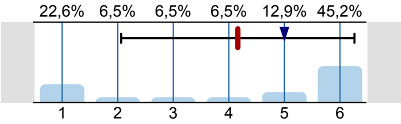

trifft voll zu

n=31
mw=4,2
md=5
s=2,1

6.2) Arbeitsplatz-basierte Prüfungen sind hilfreiche Werkzeuge um Kompetenzen zu prüfen.

trifft gar nicht zu 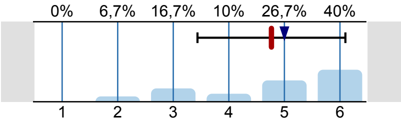

trifft voll zu

n=30
mw=4,8
md=5
s=1,3

6.3) Ich habe Mini-CEX und DOPS regelmäßig durchgeführt.

trifft gar nicht zu 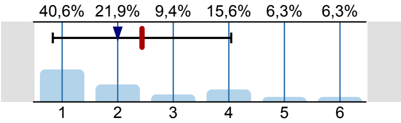

trifft voll zu

n=32
mw=2,4
md=2
s=1,6

6.4) Mini-CEX und DOPS sind effektive Hilfsmittel für den Kompetenzerwerb.

trifft gar nicht zu 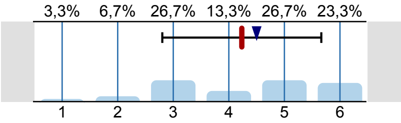

trifft voll zu

n=30
mw=4,2
md=4,5
s=1,4

6.5) Das strukturierte Feedback meiner Mentorin/ meines Mentors empfand ich als hilfreich.

trifft gar nicht zu 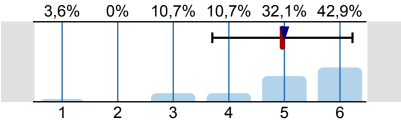

trifft voll zu

n=28
mw=5
md=5
s=1,3

6.6) ...war eine neue Erfahrung für mich.

trifft gar nicht zu 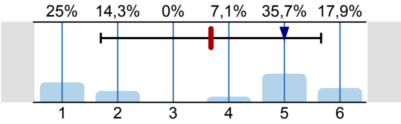

trifft voll zu

n=28
mw=3,7
md=5
s=2

6.7) ...half mir effektiv besser zu werden.

trifft gar nicht zu 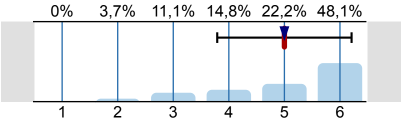

trifft voll zu

n=27
mw=5
md=5
s=1,2

6.8) ...half mir meine Fähig- und Fertigkeiten besser einzuschätzen.

trifft gar nicht zu 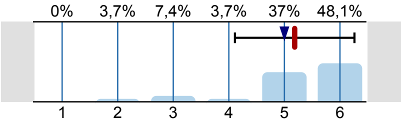

trifft voll zu

n=27
mw=5,2
md=5
s=1,1

6.9) ...empfand ich als unangenehm.

trifft gar nicht zu 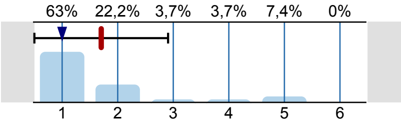

trifft voll zu

n=27
mw=1,7
md=1
s=1,2

Nr.	Frage	Linker Pol	1	2	3	4	5	6	Rechter Pol	Statistik
6.10)	...empfand ich als ehrlich.	trifft gar nicht zu	0%	0%	3,7%	7,4%	25,9%	63%	trifft voll zu	n=27 mw=5,5 md=6 s=0,8
6.11)	...war unnötig.	trifft gar nicht zu	80,8%	11,5%	7,7%	0%	0%	0%	trifft voll zu	n=26 mw=1,3 md=1 s=0,6
6.12)	Ich hätte mir mehr strukturiertes Feedback gewünscht.	trifft gar nicht zu	17,2%	17,2%	13,8%	10,3%	24,1%	17,2%	trifft voll zu	n=29 mw=3,6 md=4 s=1,8
6.13)	**Gesamtnote zu *Formative Prüfung und Feedback* (Schulnoten)**	1	28,6%	39,3%	25%	3,6%	3,6%	0%	6	n=28 mw=2,1 md=2 s=1

7. Gesamtevaluation

Nr.	Frage	Linker Pol	1	2	3	4	5	6	Rechter Pol	Statistik
7.1)	Ich bin mit meinem Lernerfolg zufrieden.	trifft gar nicht zu	0%	0%	9,4%	34,4%	34,4%	21,9%	trifft voll zu	n=32 mw=4,7 md=5 s=0,9
7.2)	Das Lernklima empfand ich als angenehm.	trifft gar nicht zu	0%	0%	3,1%	15,6%	34,4%	46,9%	trifft voll zu	n=32 mw=5,3 md=5 s=0,8
7.3)	Ich habe mich respektiert und wohl gefühlt.	trifft gar nicht zu	0%	0%	9,7%	9,7%	45,2%	35,5%	trifft voll zu	n=31 mw=5,1 md=5 s=0,9
7.4)	Auf den Teilbereich Anästhesiologie des mündlichen Examens (M3) fühle ich mich nach dem Tertial gut vorbereitet.	trifft gar nicht zu	3,1%	3,1%	12,5%	15,6%	53,1%	12,5%	trifft voll zu	n=32 mw=4,5 md=5 s=1,2
7.5)	Bei einem Berufseinstieg als Anästhesistin/ Anästhesist würde ich mich gut vorbereitet fühlen.	trifft gar nicht zu	3,2%	16,1%	9,7%	19,4%	29%	22,6%	trifft voll zu	n=31 mw=4,2 md=5 s=1,5
7.6)	Mein Tertial in der Anästhesiologie und Intensivtherapie würde ich weiter empfehlen.	trifft gar nicht zu	0%	0%	6,3%	9,4%	12,5%	71,9%	trifft voll zu	n=32 mw=5,5 md=6 s=0,9
7.7)	Interessierten würde ich den Berufsstart an der hiesigen Klinik und Poliklinik für Anästhesiologie und Intensivtherapie empfehlen.	trifft gar nicht zu	0%	0%	6,3%	18,8%	40,6%	34,4%	trifft voll zu	n=32 mw=5 md=5 s=0,9
7.8)	Ich könnte mir vorstellen mich an der hiesigen Klinik für Anästhesiologie und Intensivtherapie zu bewerben.	trifft gar nicht zu	6,3%	15,6%	3,1%	9,4%	18,8%	46,9%	trifft voll zu	n=32 mw=4,6 md=5 s=1,7
7.9)	**Gesamtnote des PJ-Tertials (Schulnoten)**	1	43,8%	43,8%	12,5%	0%	0%	0%	6	n=32 mw=1,7 md=2 s=0,7

Profillinie

Teilbereich: Anästhesiologie
Name der/des Lehrenden: Arbeitsgruppe Medizindidaktik
Titel der Lehrveranstaltung: (Name der Umfrage) Praktisches Jahr in der KAI

Verwendete Werte in der Profillinie: Mittelwert

2.1)	...erlebte ich mich als neugierig und wissbegierig.	fast nie	sehr häufig	n=32	mw=3,6	md=4,0	s=0,5
2.2)	...machte mir das Arbeiten Spaß.	fast nie	sehr häufig	n=32	mw=3,5	md=4,0	s=0,6
2.3)	...war ich von der Sache so fasziniert, dass ich alles um mich herum vergaß.	fast nie	sehr häufig	n=32	mw=2,6	md=3,0	s=0,9
2.4)	...fand ich das Lernen richtig spannend.	fast nie	sehr häufig	n=32	mw=3,3	md=3,0	s=0,7
2.5)	...hatte ich das Gefühl, mich kaum von der Sache lösen zu können.	fast nie	sehr häufig	n=32	mw=2,5	md=2,5	s=0,8
2.6)	...hatte ich den Eindruck nur aufgrund des mündlichen Examens (M3) zu lernen.	fast nie	sehr häufig	n=32	mw=1,3	md=1,0	s=0,7
2.7)	...empfand ich das Lernen als anstrengend.	fast nie	sehr häufig	n=32	mw=2,0	md=2,0	s=0,9
2.8)	...hatte ich das Gefühl mich zum Arbeiten zwingen zu müssen.	fast nie	sehr häufig	n=32	mw=1,5	md=1,0	s=0,6
2.9)	...merkte ich selbst, was ich kann bzw. was ich noch nicht kann.	fast nie	sehr häufig	n=32	mw=3,6	md=4,0	s=0,6
2.10)	...fühlte ich mich aktiv.	fast nie	sehr häufig	n=32	mw=3,1	md=3,0	s=0,8
2.11)	...ergaben sich aus dem klinischen Alltag Probleme, mit denen ich mich eingehender beschäftigen möchte.	fast nie	sehr häufig	n=32	mw=3,5	md=4,0	s=0,6
2.12)	...hatte ich den Eindruck, meinen Lernerfolg selbst steuern zu können.	fast nie	sehr häufig	n=32	mw=3,0	md=3,0	s=0,7
2.13)	...fühlte ich mich ernst genommen.	fast nie	sehr häufig	n=32	mw=3,3	md=3,0	s=0,7
2.14)	...fühlte ich mich stark kontrolliert.	fast nie	sehr häufig	n=32	mw=2,0	md=2,0	s=0,8
2.15)	...hatte ich das Gefühl, etwas zu tun, was ich auch selber tun wollte.	fast nie	sehr häufig	n=32	mw=3,4	md=3,5	s=0,6
2.16)	...hatte ich das Gefühl, Entscheidungsspielräume zu haben.	fast nie	sehr häufig	n=32	mw=2,8	md=3,0	s=1,0
3.1)	Für meinen Einstieg ins Tertial benötige ich mehr Unterstützung.	trifft gar nicht zu	trifft voll zu	n=32	mw=2,7	md=2,5	s=1,5
3.2)	Die Teilnahme an Diensten empfand ich als sinnvoll.	trifft gar nicht zu	trifft voll zu	n=28	mw=5,4	md=6,0	s=0,8
3.3)	Im Operationssaal war das Tertial gut strukturiert.	trifft gar nicht zu	trifft voll zu	n=32	mw=4,0	md=4,5	s=1,4
3.4)	Im Operationssaal war das Tertial fachlich anspruchsvoll.	trifft gar nicht zu	trifft voll zu	n=32	mw=4,8	md=5,0	s=1,1
3.5)	Auf der Intensivstation war das Tertial gut strukturiert.	trifft gar nicht zu	trifft voll zu	n=32	mw=4,8	md=5,0	s=1,3
3.6)	Auf der Intensivstation war das Tertial fachlich anspruchsvoll.	trifft gar nicht zu	trifft voll zu	n=32	mw=5,7	md=6,0	s=0,6
3.7)	Das Tertial bot mir ausreichend Zeit für Selbststudium.	trifft gar nicht zu	trifft voll zu	n=32	mw=3,5	md=4,0	s=1,5
3.8)	Meine Ansprechpartner in der Klinik waren klar definiert.	trifft gar nicht zu	trifft voll zu	n=32	mw=5,6	md=6,0	s=0,7
3.9)	Die Organisation von Hospitationstagen (Palliativmedizin, Notfallmedizin, Schmerzmedizin) war einfach.	trifft gar nicht zu	trifft voll zu	n=30	mw=5,4	md=6,0	s=1,1
3.10)	Der Wechsel zwischen Operationsaal und Intensivstation verlief reibungslos.	trifft gar nicht zu	trifft voll zu	n=32	mw=5,4	md=6,0	s=1,0

Nr.	Item	Linker Pol	Rechter Pol	n	mw	md	s
3.11)	Auf meine persönlichen Wünsche wurde eingegangen.	trifft gar nicht zu	trifft voll zu	n=32	mw=5,6	md=6,0	s=0,7
3.12)	Bei Problemen wurde mir zeitnah geholfen.	trifft gar nicht zu	trifft voll zu	n=32	mw=5,5	md=6,0	s=0,8
3.13)	Ich fühlte mich als Teil des Behandlungteams.	trifft gar nicht zu	trifft voll zu	n=31	mw=4,6	md=5,0	s=1,6
3.14)	**Gesamtnote *Ablauf und Struktur* (Schulnoten)**	1	6	n=31	mw=1,7	md=2,0	s=0,6
4.1)	...habe ich immer besucht.	trifft gar nicht zu	trifft voll zu	n=32	mw=5,0	md=5,0	s=0,8
4.2)	...war praxisbezogen und klinikalltagsrelevant.	trifft gar nicht zu	trifft voll zu	n=32	mw=5,3	md=5,0	s=0,8
4.3)	...würde ich weiterempfehlen.	trifft gar nicht zu	trifft voll zu	n=32	mw=5,6	md=6,0	s=0,8
4.4)	...waren thematisch sinnvoll gewählt.	trifft gar nicht zu	trifft voll zu	n=32	mw=5,4	md=6,0	s=0,8
4.5)	...waren sinnvoll aufeinander abgestimmt.	trifft gar nicht zu	trifft voll zu	n=32	mw=4,7	md=5,0	s=1,0
4.6)	...haben mein Verständnis und Lernen gefördert.	trifft gar nicht zu	trifft voll zu	n=31	mw=5,5	md=6,0	s=0,6
4.7)	...fanden regelmäßig statt.	trifft gar nicht zu	trifft voll zu	n=32	mw=4,4	md=4,5	s=1,1
4.8)	Die Referntinnen und Referenten hatten Spaß am Lehren.	trifft gar nicht zu	trifft voll zu	n=32	mw=5,3	md=5,0	s=0,7
4.9)	...war gut strukturiert.	trifft gar nicht zu	trifft voll zu	n=16	mw=3,3	md=3,5	s=2,1
4.10)	...hatte einen persönlichen Mehrwert für mich.	trifft gar nicht zu	trifft voll zu	n=16	mw=3,9	md=4,5	s=2,2
4.11)	...sollte häufiger angeboten werden.	trifft gar nicht zu	trifft voll zu	n=21	mw=5,0	md=6,0	s=1,7
4.12)	Nach dem Simulationstraining fühlte ich mich auf eine Notfallsituation besser vorbereitet.	trifft gar nicht zu	trifft voll zu	n=16	mw=3,4	md=3,5	s=2,1
4.13)	**Gesamtnote *PJ-Seminar und Simulationstraining* (Schulnoten)**	1	6	n=31	mw=2,1	md=2,0	s=0,7
5.1)	...erleichterte mir den Einstieg in das Tertial.	trifft gar nicht zu	trifft voll zu	n=32	mw=5,5	md=6,0	s=1,2
5.2)	...ist lehrbegeistert.	trifft gar nicht zu	trifft voll zu	n=31	mw=5,6	md=6,0	s=0,7
5.3)	...ist eine gute Lehrerin/ein guter Lehrer.	trifft gar nicht zu	trifft voll zu	n=31	mw=5,6	md=6,0	s=0,7
5.4)	...ist für mich ein Vorbild.	trifft gar nicht zu	trifft voll zu	n=31	mw=5,2	md=6,0	s=1,2
5.5)	...gab mir effektives Feedback.	trifft gar nicht zu	trifft voll zu	n=31	mw=5,5	md=6,0	s=1,1
5.6)	...war fachlich kompetent.	trifft gar nicht zu	trifft voll zu	n=31	mw=5,6	md=6,0	s=0,6
5.7)	...motivierte mich und förderte mein eigenständiges Lernen.	trifft gar nicht zu	trifft voll zu	n=31	mw=5,4	md=6,0	s=1,2
5.8)	Das Mentoring-Programm verhalf mir zu einem besseren Lernerfolg.	trifft gar nicht zu	trifft voll zu	n=31	mw=5,3	md=6,0	s=1,1
5.9)	Der Ausbildungsstand (Arzt in Weiterbildung, Facharzt) meiner Mentorin/meines Mentors ist mir wichtig.	trifft gar nicht zu	trifft voll zu	n=32	mw=3,5	md=4,0	s=1,8
5.10)	Ich empfinde die Kontaktaufnahme zum Tertialbeginn durch das Mentoring-Team als unnötig.	trifft gar nicht zu	trifft voll zu	n=32	mw=1,3	md=1,0	s=0,7
5.11)	Ich fühlte mich durch meine Mentorin/mein Mentor gut begleitet.	trifft gar nicht zu	trifft voll zu	n=32	mw=5,0	md=5,5	s=1,3
5.12)	Im Nachhinein würde ich mir eine engmaschigere Betreuung wünschen.	trifft gar nicht zu	trifft voll zu	n=32	mw=3,6	md=4,0	s=1,6
5.13)	**Gesamtnote zu *Mentoring* (Schulnoten)**	1	6	n=31	mw=1,5	md=1,0	s=0,8

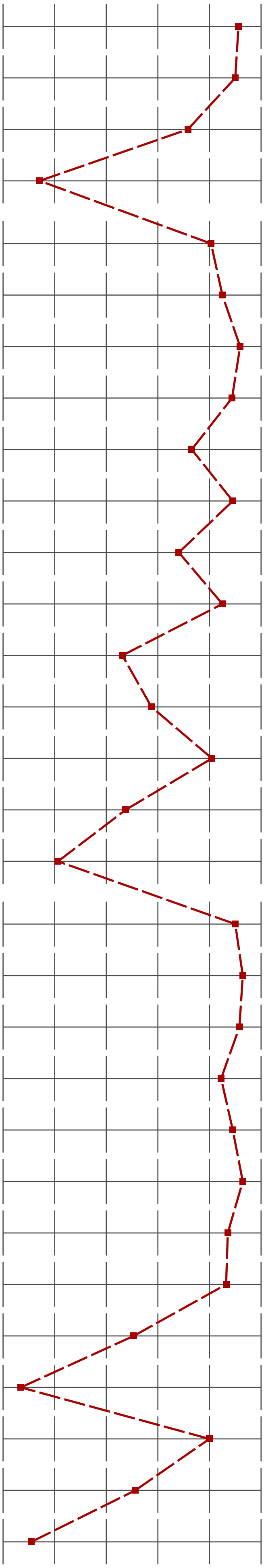

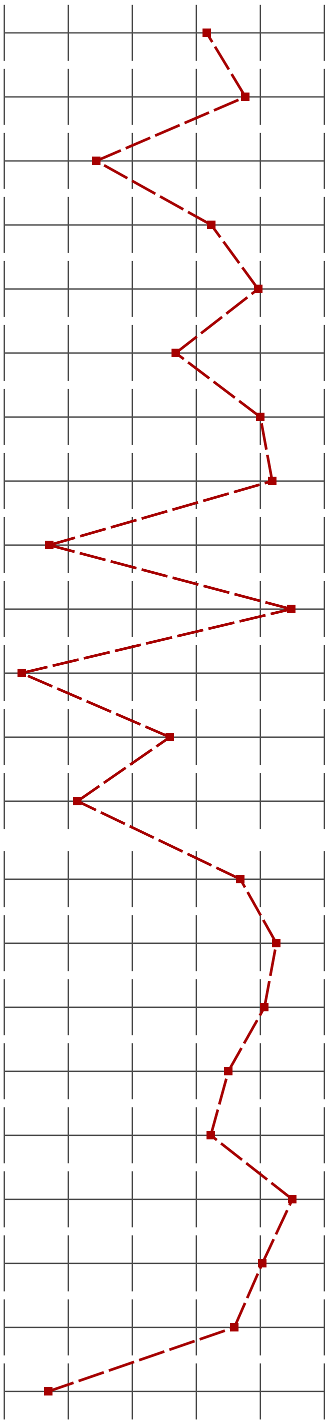

6.1)	Arbeitsplatz-basierte Prüfungen sind mir neu.	trifft gar nicht zu	trifft voll zu	n=31	mw=4,2	md=5,0	s=2,1
6.2)	Arbeitsplatz-basierte Prüfungen sind hilfreiche Werkzeuge um Kompetenzen zu prüfen.	trifft gar nicht zu	trifft voll zu	n=30	mw=4,8	md=5,0	s=1,3
6.3)	Ich habe Mini-CEX und DOPS regelmäßig durchgeführt.	trifft gar nicht zu	trifft voll zu	n=32	mw=2,4	md=2,0	s=1,6
6.4)	Mini-CEX und DOPS sind effektive Hilfsmittel für den Kompetenzerwerb.	trifft gar nicht zu	trifft voll zu	n=30	mw=4,2	md=4,5	s=1,4
6.5)	Das strukturierte Feedback meiner Mentorin/ meines Mentors empfand ich als hilfreich.	trifft gar nicht zu	trifft voll zu	n=28	mw=5,0	md=5,0	s=1,3
6.6)	...war eine neue Erfahrung für mich.	trifft gar nicht zu	trifft voll zu	n=28	mw=3,7	md=5,0	s=2,0
6.7)	...half mir effektiv besser zu werden.	trifft gar nicht zu	trifft voll zu	n=27	mw=5,0	md=5,0	s=1,2
6.8)	...half mir meine Fähig- und Fertigkeiten besser einzuschätzen.	trifft gar nicht zu	trifft voll zu	n=27	mw=5,2	md=5,0	s=1,1
6.9)	...empfand ich als unangenehm.	trifft gar nicht zu	trifft voll zu	n=27	mw=1,7	md=1,0	s=1,2
6.10)	...empfand ich als ehrlich.	trifft gar nicht zu	trifft voll zu	n=27	mw=5,5	md=6,0	s=0,8
6.11)	...war unnötig.	trifft gar nicht zu	trifft voll zu	n=26	mw=1,3	md=1,0	s=0,6
6.12)	Ich hätte mir mehr strukturiertes Feedback gewünscht.	trifft gar nicht zu	trifft voll zu	n=29	mw=3,6	md=4,0	s=1,8
6.13)	**Gesamtnote zu *Formative Prüfung und Feedback* (Schulnoten)**	1	6	n=28	mw=2,1	md=2,0	s=1,0
7.1)	Ich bin mit meinem Lernerfolg zufrieden.	trifft gar nicht zu	trifft voll zu	n=32	mw=4,7	md=5,0	s=0,9
7.2)	Das Lernklima empfand ich als angenehm.	trifft gar nicht zu	trifft voll zu	n=32	mw=5,3	md=5,0	s=0,8
7.3)	Ich habe mich respektiert und wohl gefühlt.	trifft gar nicht zu	trifft voll zu	n=31	mw=5,1	md=5,0	s=0,9
7.4)	Auf den Teilbereich Anästhesiologie des mündlichen Examens (M3) fühle ich mich nach dem Tertial gut vorbereitet.	trifft gar nicht zu	trifft voll zu	n=32	mw=4,5	md=5,0	s=1,2
7.5)	Bei einem Berufseinstieg als Anästhesistin/ Anästhesist würde ich mich gut vorbereitet fühlen.	trifft gar nicht zu	trifft voll zu	n=31	mw=4,2	md=5,0	s=1,5
7.6)	Mein Tertial in der Anästhesiologie und Intensivtherapie würde ich weiter empfehlen.	trifft gar nicht zu	trifft voll zu	n=32	mw=5,5	md=6,0	s=0,9
7.7)	Interessierten würde ich den Berufsstart an der hiesigen Klinik und Poliklinik für Anästhesiologie und Intensivtherapie	trifft gar nicht zu	trifft voll zu	n=32	mw=5,0	md=5,0	s=0,9
7.8)	Ich könnte mir vorstellen mich an der hiesigen Klinik für Anästhesiologie und Intensivtherapie zu bewerben.	trifft gar nicht zu	trifft voll zu	n=32	mw=4,6	md=5,0	s=1,7
7.9)	**Gesamtnote des PJ-Tertials (Schulnoten)**	1	6	n=32	mw=1,7	md=2,0	s=0,7
